# Visible-Light-Promoted Iridium(III)-Catalyzed Acceptorless Dehydrogenation of N-Heterocycles at Room Temperature

**DOI:** 10.1021/acscatal.2c01224

**Published:** 2022-05-10

**Authors:** Carmen Mejuto, Laura Ibáñez-Ibáñez, Gregorio Guisado-Barrios, Jose A. Mata

**Affiliations:** † Institute of Advanced Materials (INAM), Centro de Innovación en Química Avanzada (ORFEO-CINQA), Universitat Jaume I, Avda. Sos Baynat s/n, 12006 Castellón, Spain; ‡ Departamento de Química Inorgánica. Instituto de Síntesis Química y Catálisis Homogénea (ISQCH), CSIC-Universidad de Zaragoza, 50009 Zaragoza, Spain

**Keywords:** photocatalysis, iridium, N-heterocycles, hydrogenation, dehydrogenation, LOHCs, hydrogen storage

## Abstract

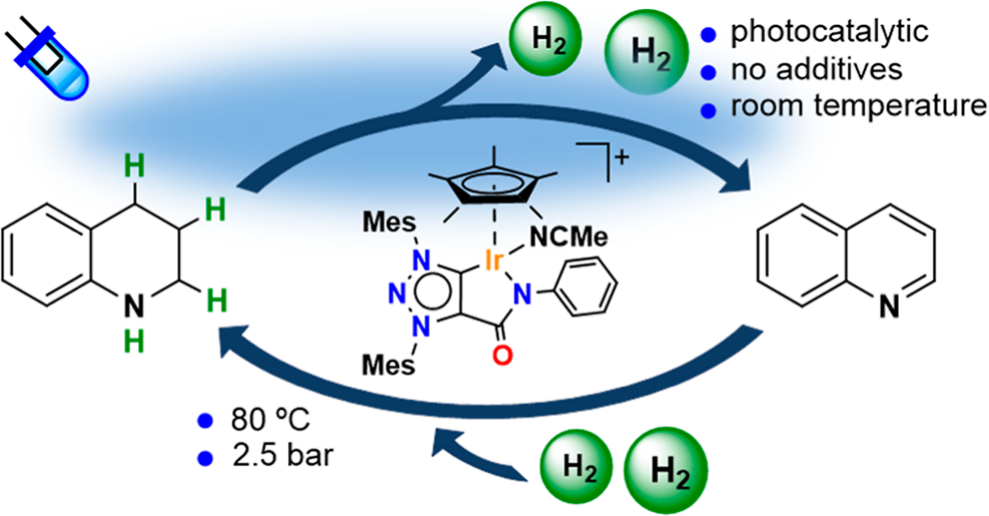

An effective visible-light-promoted iridium(III)-catalyzed hydrogen production from N-heterocycles is described. A single iridium complex constitutes the photocatalytic system playing a dual task, harvesting visible-light and facilitating C–H cleavage and H_2_ formation at room temperature and without additives. The presence of a chelating C–N ligand combining a mesoionic carbene ligand along with an amido functionality in the Ir^III^ complex is essential to attain the photocatalytic transformation. Furthermore, the Ir^III^ complex is also an efficient catalyst for the thermal reverse process under mild conditions, positioning itself as a proficient candidate for liquid organic hydrogen carrier technologies (LOHCs). Mechanistic studies support a light-induced formation of H_2_ from the Ir–H intermediate as the operating mode of the iridium complex.

## Introduction

**Figure 1:**
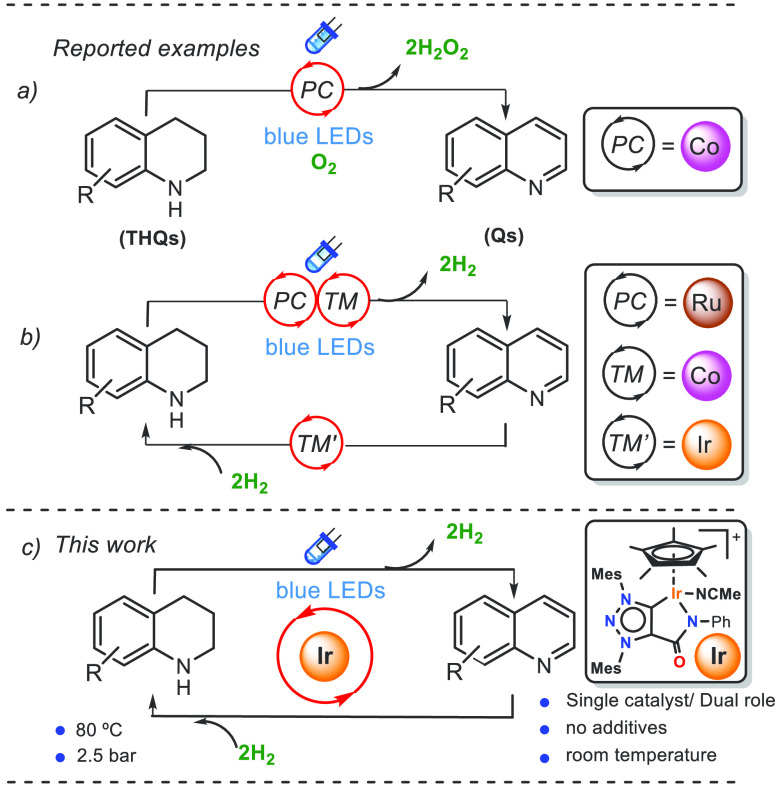
Sketch illustrating the concept of a single iridium catalyst in photodehydrogenation and thermal hydrogenation of N-heterocycles.

The catalytic (de)hydrogenation of N-heterocycles involving H_2_ is a fundamental organic transformation being present in the manufacturing of multistep synthesis of drugs and biologically relevant molecules.^1−4^ Notably, the use of N-heterocycles has recently gained considerable attention for storage of hydrogen as liquid organic hydrogen carrier (LOHC) which is based on catalytic (de)hydrogenations of organic substrates.^5−8^ Still, development of efficient catalysts for hydrogen-storage has proven challenging, being subjected to fulfill a series of technical and practical prerequisites to become a disruptive technology.^9−13^ One of the most constraining factors is the temperature at which the hydrogen is recovered from the organic carrier (90–300 °C).^14,15^ Thermodynamically, dehydrogenation of organic molecules to release H_2_ is an uphill process requiring high temperatures.^16,17^ To overcome this energetic barrier, the use of visible light (400–700 nm) has emerged as a valuable and cleaner alternative.^18−20^ Specially, because photon energies exhibit values superior or within the range of those energy barriers found for many catalyzed reactions thermally activated. Therefore, many efforts have been directed to provide viable alternatives. For instance, Baskar et al. described an efficient cobalt-based homogeneous water soluble photoredox catalyst (PC) for the oxidative dehydrogenation of tetrahydroquinolines (THQs) affording the corresponding quinolines (Qs) and hydrogen peroxide under mild conditions (Figure 1a).^21^ In this case, the metal complex CoPc(SO_3_Na)_4_ (Pc = phthalocyanine) acts as a photocatalyst (PC) absorbing visible light, inducing a single electron transfer process, which is accountable for the success of the reaction. Yet, the use of O_2_ as an oxidant precludes the formation of H_2_. In contrast, Kanai et al. reported acceptorless photodehydrogenation of N-heterocycles using a hybrid catalyst comprising an acridinium photoredox and a palladium metal catalyst.^22^ In parallel, the groups of Li and Balaraman have recently reported the acceptorless dehydrogenation of N-heterocycles using visible-light catalyzed by an efficient sophisticated photoredox catalytic system comprising a ruthenium-based photosensitizer [Ru(bpy)_3_]^2+^ and a cobalt complex [Co(dmgH)_2_PyCl] (dmgH = dimethylglyoximate) (Figure 1b). Besides that, the system reported by Li and co-workers includes an additional dinuclear iridium catalyst [{Ir(Cp*)(Cl)}_2_(thbpym)] bearing (thbpym = 4,4′,6,6′-tetrahydroxy-2,2′-bipyrimidine) as a bridging ligand to efficiently catalyze the reverse process (i.e., hydrogenation of N-heterocycles).^23−25^ Notably, the availability of a reversible dehydrogenation–hydrogenation process was used to prove the great potential of this strategy in future applications for H_2_ storage materials. Nevertheless, further improvements such as finding a single catalyst capable of carrying out both transformations are highly desirable.^26−30^

In this context, visible-light-induced catalysis, where a single metal complex absorbs visible light and contributes to the bond breaking/bond forming process has merged as an alternative to classic photoredox catalysts.^31−34^ For instance, Pitman and Miller recently reported the light-triggered formic acid dehydrogenation catalyzed by [Cp*Ir(bpy)(Cl)]Cl (bpy = 2,2′-bipyridine),^35^ whereas Chirik et al. described the photo-induced hydrogenation and hydrogen atom transfer of weak chemical bonds using iridium hydride species.^36,37^ Likewise, Baslé et al. unveiled a single Rh(I) complex bearing an N-heterocyclic carbene ligand (NHC), which efficiently catalyzes the visible-light-promoted regioselective borylation of aromatic C–H bonds.^38^ The key to the success for the latest was the chelating nature of the NHC-carboxylate ligand conferring stability to the metal complex and delivering exceptional photocatalytic activities. With regards to (de)hydrogenation of N-heterocycles, despite great strides in the field, to the best of our knowledge, the availability of a single catalyst capable of releasing H_2_ from N-heterocycles at low temperatures has not yet been uncovered. Founded on these precedents, our experience on the use of NHCs and mesoionic triazolylidenes as ancillary ligands (MICs), and its application for transition metal-based catalytic hydrogen generation,^39−42^ we hypothesized that a single MIC–Ir^III^ having an MIC-amido ligand could possibly be used for visible light-assisted dehydrogenation reactions. Herein, in this work, we report the acceptorless photodehydrogenation of N-heterocycles at room temperature promoted by visible-light using a single MIC–Ir^III^ complex catalyst without additives. In addition, the same iridium(III) complex is an efficient catalyst toward the reverse reaction carried out thermally under mild conditions (Figure 1c).

## Results and Discussion

**Scheme 1:**
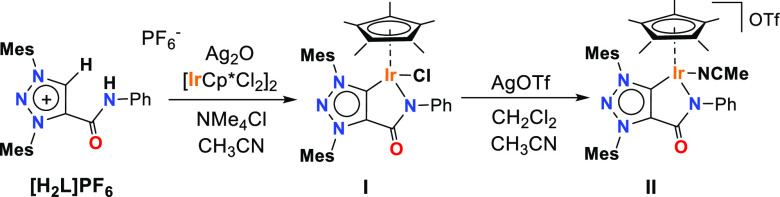
Synthesis of MIC–Ir^III^ Complexes **I** and **II**

We initiated our studies by preparing iridium complexes with a chelating MIC ligand. The precursor 1,3-dimesityl-1*H*-1,2,3-triazolium ligand salt **[H**_**2**_**L]PF**_**6**_ bearing a pendant amide functional group was obtained by the cycloaddition of *N*-phenylpropiolamide and 1,3-bis(2,4,6-trimethylphenyl)triaz-1-ene (Scheme 1).^43,44^ The neutral MIC–Ir^III^ complex **I** containing a tethered amido-MIC ligand was obtained in a 95% yield as a brown-orange solid from a one-pot reaction via transmetallation from the silver derivative and [IrCp*Cl_2_]_2_ in the presence of tetramethylammonium chloride as the halide source. The coordination of the iridium center to the organic fragment was confirmed by the absence of the two acidic protons of the ligand salt in the ^1^H NMR at 9.29 and 9.71 ppm. The synthesis of the related cationic MIC–Ir^III^ complex **II** was accomplished by using silver triflate as halide abstraction in a dichloromethane/acetonitrile mixture (15:2). Complex **II** was isolated as a brown solid in 76% yield. All the compounds were fully characterized by ^1^H and ^13^C NMR spectroscopy and HR-MS spectrometry and the photochemical properties were analyzed by visible light absorption (Supporting Information). The UV–vis spectra for complexes **I** and **II** were registered in methanol, and an absorption band was observed at 365 nm. The cationic complex **II** exhibited higher absorbance than the neutral derivative **I**. Notably, albeit a solution of MIC–Ir^III^ complex **II** does not absorb strongly at wavelengths greater than 400 nm, there is an overlap between its absorption spectrum and the emission spectrum of the blue-LED light source (Figure S25).

We continued our studies by exploring the feasibility of conducting the visible-light-induced catalytic dehydrogenation of diverse N-heterocycles using MIC–Ir^III^ complexes (**I** and **II**) under different reaction conditions (Table 1). Blank experiments using 1,2,3,4-tetrahydroquinoline (THQ) or 8-methyl-THQ as substrates either in toluene or methanol irradiated with 2 × 50 W blue LEDs (λ_max_ = 455 nm) for 18 h in the absence of MIC–Ir^III^ complexes did not proceed (entries 1–4). In contrast, when the reaction was performed under the same reaction conditions but in the presence of complex **I** (2.0 mol %), dehydrogenated products, quinoline and 6-MeO-THQ, were observed in a low 8 and 35% yields, respectively (entries 5 and 6). Interestingly, when MIC–Ir complex **II** was used instead, the dehydrogenated products were obtained in 73 and 80% yields, respectively (entries 7 and 8). The use of non-polar solvents, such as toluene, had a detrimental effect in the dehydrogenation of THQs and lower yields were obtained (entries 9 and 10). In view of these results, methanol and complex **II** were selected as a suitable combination for the light-induced dehydrogenation of THQs. Nevertheless, independently of the solvent used, the acceptorless photodehydrogenation process requires a complete exclusion of air from the reaction vessel and solvents. We did not observe product formation or in low yields when the photodehydrogenation was performed under air or using non-deoxygenated solvents. For this reason, solvents were deoxygenated by saturation with nitrogen gas using the freeze–pump–thaw methodology. The light-promoted catalytic dehydrogenation of THQ produces quinoline (Q) without additives at room temperature with the concomitant release of two molecules of H_2_. The presence of molecular hydrogen was qualitatively confirmed using a mass spectrometer (Figure S18). Acceptorless dehydrogenation of N-heterocycles is an uphill process and requires the removal of H_2_ from the reaction media to proceed efficiently. Consequently, acceptorless dehydrogenation reactions are very sensitive to pressure variations; in fact, the reaction does not evolve when carried out in a closed system. These experiments support an acceptorless pathway rather than an aerobic oxidation for the photodehydrogenation of N-heterocycles using MIC–Ir^III^ complexes. To find the optimal reaction conditions, we also tested the influence of the incident light. As a control experiment, when the reaction was carried in the dark, no product was formed, confirming the need of visible light for the reaction to proceed (entries 11 and 12). On the contrary, the yield of the reaction increased to 89% (ethanol) or 95% (methanol) when a light source of blue LEDs (λ_max_ = 455 nm) was used as the light source (entries 13 and 14). Attempts to use a less energetic light source, green LEDs (λ_max_ = 529 nm) or red LEDs (λ_max_ = 635 nm), resulted detrimental to the performance of the photocatalytic system and low yields of dehydrogenated products were obtained (entries 15 and 16). Similarly, while 2-methylindoline could be dehydrogenated affording 2-methylindole in 95% yield using blue LED irradiation, green light produced less than 5% of the product (entries 17 and 18). Photocatalytic dehydrogenation of N-heterocycles under different conditions highlighted the importance of blue light irradiation, methanol as solvent, and the presence of a labile ligand in the coordination sphere of the MIC–Ir^III^ complex.

**Table 1:**
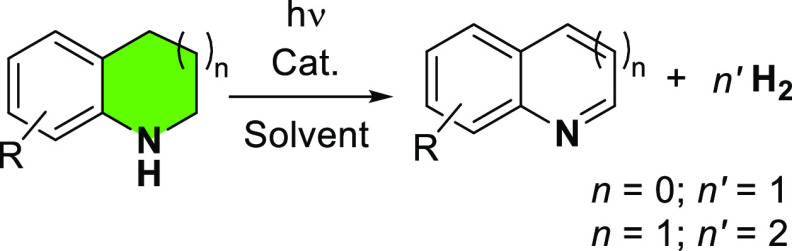

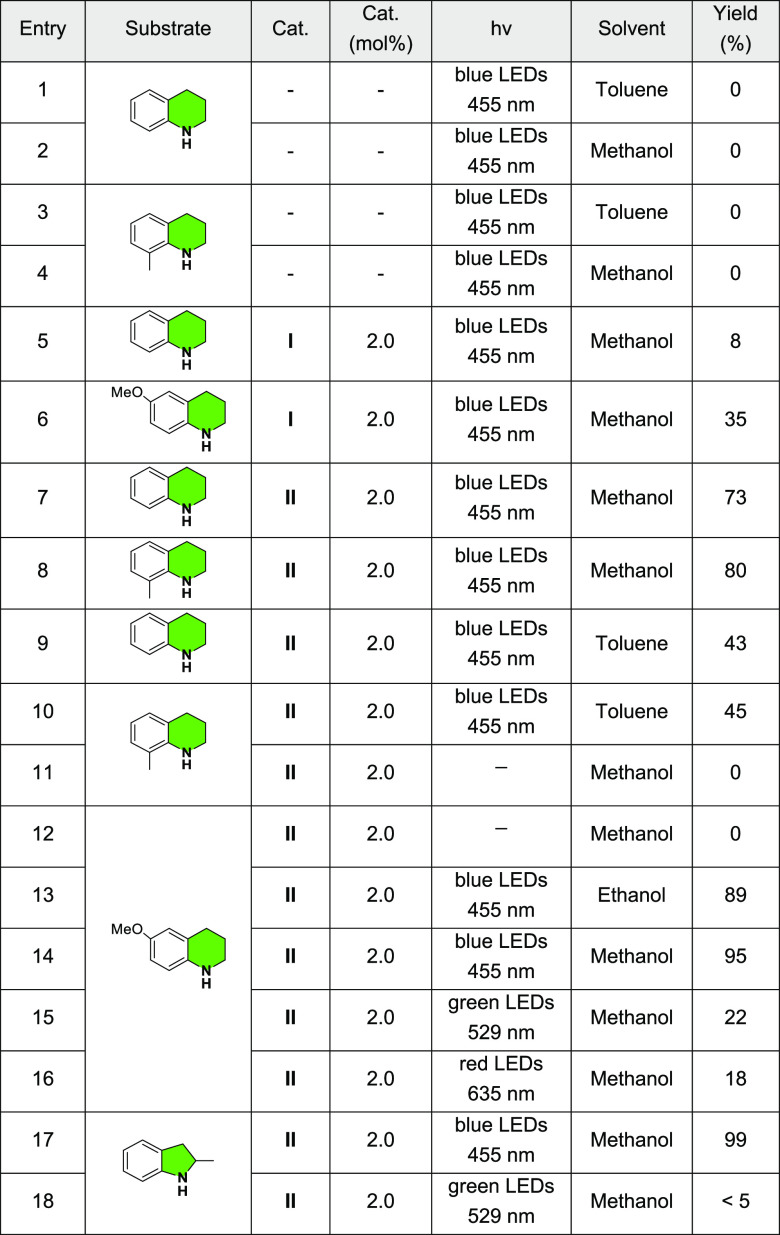
Optimization of Reaction Conditions in the Photodehydrogenation of N-Heterocycles^a^

a Reaction conditions: anaerobic conditions under nitrogen, substrate (0.2 mmol), catalyst, solvent (2 mL, dry and deoxygenated), LED irradiation for 18 h at room temperature. Product formation (yield) obtained by GC/FID using 1,3,5-trimethoxybenzene as an internal standard.

Next, we investigated the substrate scope and limitations (Table 2). The MIC–Ir^III^ complex **II** acts as an efficient catalyst in the dehydrogenation of six- and five-membered ring N-heterocycles. Importantly, dehydrogenation occurs at room temperature induced by blue LED irradiation. In fact, the reaction did not proceed when dehydrogenation of these substrates was tested in the dark. It is of particular interest that no additives (bases, photosensitizers, initiators, etc.) are required in the dehydrogenation, increasing the atom efficiency and the E-factor of this photocatalytic transformation versus traditional systems. Many photocatalytic transformations recently described induce the activation of inert bonds, but the number of additives hampers the sustainability of the process. In the present study, a variety of N-heterocycles with different substituents are photodehydrogenated in quantitative yields with the only presence of complex **II**. N-Heterocycles such as THQ or substituted THQ with one electron-donating group (compounds **1H–5H**) can be converted into the related dehydrogenated derivatives with the concomitant formation of H_2_ in high yields that range from 90–70%. In contrast, the light-promoted iridium-catalyzed dehydrogenation is less favorable for those substrates containing electron-withdrawing groups. For instance, halogen-containing substrates **6H** and **7H** produced the corresponding derivatives **6D** and **7D** in low 24 and 25% yields, respectively. The presence of a strong electron-withdrawing nitro group, **8H**, completely hampers the reaction. In the case of N-heterocycles containing five-membered groups (indole derivatives), they are easily dehydrogenated under these conditions (compounds **9D** and **10D**), although only one equivalent of H_2_ is produced. The photodehydrogenation of tetrahydroquinoxalines with different substitutions at C2 or C3 positions provided full conversion, although with low yields most probably due to ring opening decomposition reactions. For instance, tetrahydroquinoxaline **11H** provided 89% conversion but only 33% yield. The use of tetrahydroisoquinolines as substrates provided low yields with only one equivalent of H2 at the NH–C(2)H position (compounds **12–14**).

**Table 2:**
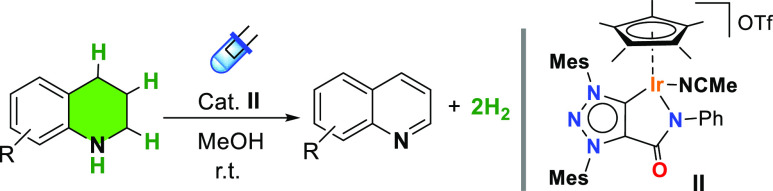

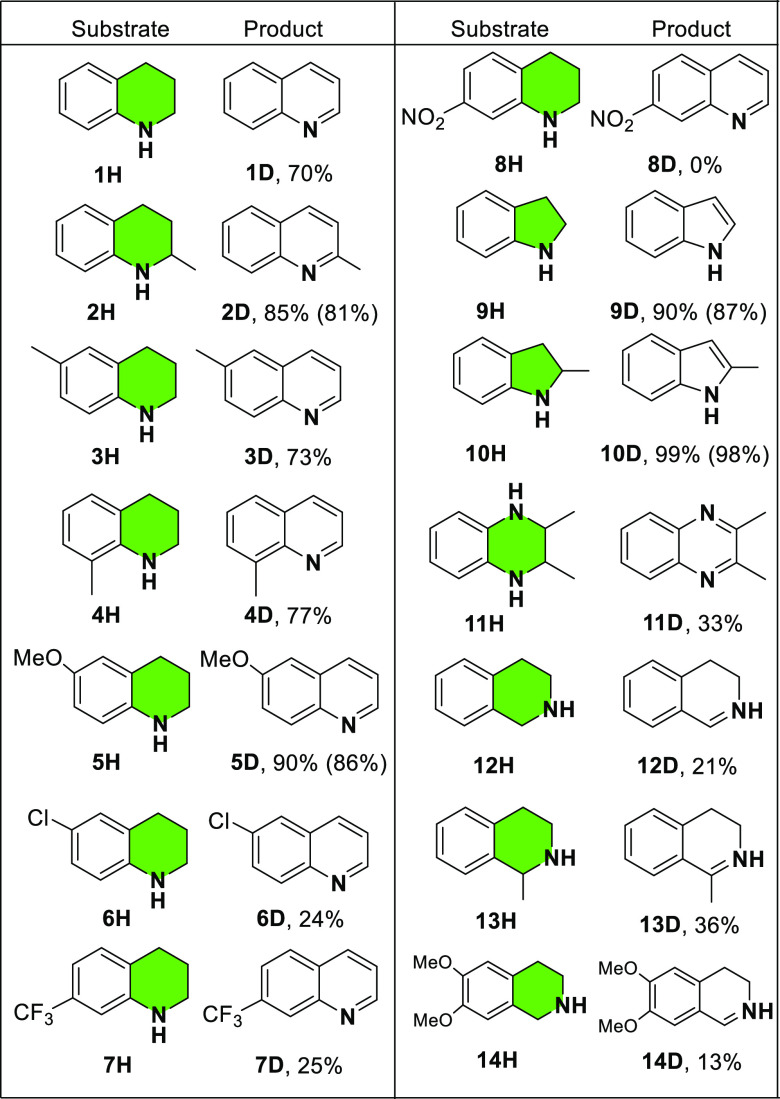
Photocatalytic Activity of Iridium Complex **II** in Dehydrogenation of N-Heterocycles^a^

a Reaction conditions: substrate (0.2 mmol), complex **II** (2.0 mol %), methanol (2 mL), and blue LEDs (455 nm) at room temperature for 18 h. Reaction yield (product formation) obtained by GC/FID using hexadecane as an internal standard. In parenthesis isolated yield. **H**, (hydrogenated); **D**, (dehydrogenated).

**Figure 2:**
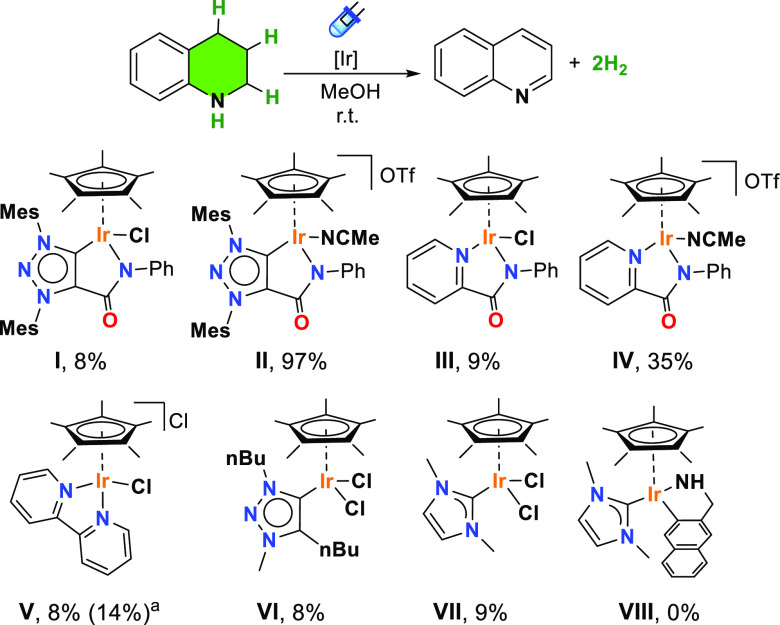
Photocatalytic activity of iridium complexes in the dehydrogenation of THQ. Conditions: substrate (0.2 mmol), methanol (2 mL), iridium(III) complexes (2.0 mol %), blue LEDs (455 nm), and 18 h at room temperature. (a) Reaction carried out in the presence of 2 equiv of AgOTf.

After establishing the optimal reaction conditions and the scope/limitations in photodehydrogenation of N-heterocycles, we evaluated the structure and ligand influence of iridium complexes. For comparison purposes, the performance of a series of neutral and cationic iridium(III) complexes bearing either chelating C–N (complexes **I–II**), N–N (complex **III–V**),^45−47^ or monodentate carbon-based ligands (complexes **VI–VIII**)^48−50^ was evaluated in the blue-LED-promoted catalytic dehydrogenation of THQ (**1H**) (Figure 2). Among them, complex MIC–Ir^III^**II**, featuring a mesoionic triazolylidene (MIC) with an amido functionality resulted the most efficient catalyst affording quantitative yields. We observed that the presence of a labile ligand such as acetonitrile favors the dehydrogenation process. For instance, the analogous complex **I**, containing a stronger coordinating ligand (Cl^–^), affords a modest 8% yield under the same reaction conditions. A similar trend but with lower yields was observed for the neutral and the cationic iridium analogues (complexes **III** and **IV**) where the carbene unit is replaced by a pyridine. Most probably, the more coordinating chloride ligand and the mild reactions conditions impedes the generation of a vacant coordination site capable of interacting with the THQ. Strikingly, the cationic iridium complex **V** containing a bipyridine ligand, displayed low activity in dehydrogenation of THQ. This fact is especially relevant because the metal complex has been successfully employed for the light-promoted dehydrogenation of formic acid.^35^ The performance of **V** in the presence of AgOTf (2 equiv) used as chloride abstractor to generate an accessible coordination site was also assessed. Still, it did not provide any significant improvement in the photocatalytic dehydrogenation of THQ and a 14% yield of quinoline was obtained. On the other hand, complexes **VI–VII** having a monodentate MIC or NHC ligand lacking the amido functionality exhibited individually poor activity, 8 and 9% yield, respectively. These complexes do not require the addition of a halide abstractor for generating a vacant coordination site under polar solvents due to a solvolysis equilibrium as we have previously observed.^51,52^ Finally, the NHC–Ir^III^ derivative **VIII** bearing a monodentate NHC and a chelating C–N ligand resulted completely inactive. From this survey, it can be stated that subtle changes in the ligand framework have a significant impact in the photocatalytic dehydrogenation reaction. The combination of a labile acetonitrile ligand with a bidentate ligand containing and amido functionality provides the best catalytic system in photodehydrogenation of N-heterocycles (complexes **II** and **IV**). Our observations are supported by recent stoichiometric hydride transfer studies carried out by Do et al. using hydride derivatives of complex **III** resulting more efficient hydride donors compared to structurally related Cp*Ir complexes, although the origin of this reactivity may also stem from the amidate functionality.^53^ At this stage, we do not discard a similar behavior or even the participation of the mesoionic carbene ligand as it has been previously observed as a result of its non-innocent character.^54,55^

**Figure 3:**
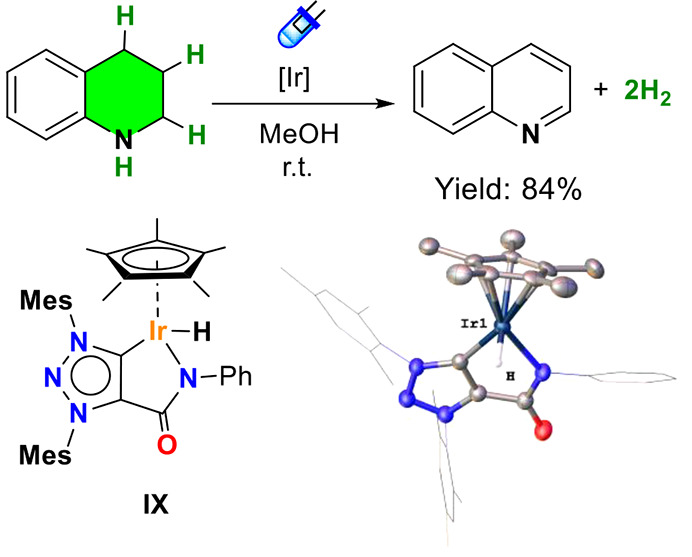
Photocatalytic activity of iridium hydride (**IX**) in the dehydrogenation of THQ. Conditions: substrate (0.2 mmol), methanol (2 mL), Ir–H (2.0 mol %), blue LEDs (455 nm), 18 h at room temperature. Structure and ORTEP diagram of Ir–H (**IX**).

Inspired by the results obtained in photodehydrogenation of THQs and the structural properties of iridium complexes, we performed a set of experiments intrigued about the operating mode of MIC-Ir^III^ complex **II**. First, we analyzed the catalytic reaction profiles and selectivity of photodehydrogenations (Figures S11–S17). The reaction profiles are characteristic of a catalytic process that occurs without an induction period and without catalyst deactivation. These results support the stability of catalyst **II** under photocatalytic conditions at least for 24 h. It should be noted that the reactions are carried out at room temperature avoiding thermal-induced deactivation processes. Reaction monitoring using ^1^H NMR did not show the formation of byproducts or substrate degradation, indicating that the conversion of THQ into the dehydrogenated products is a selective process. Then, we explored the acceptorless dehydrogenation of N-heterocycles under thermal conditions (Table S9). We started our studies using the general conditions of photodehydrogenation but without light irradiation. We did not observe product formation at room temperature or increasing the bath temperature up to 80 °C. As expected, thermal dehydrogenation required higher temperatures to proceed. The reaction of THQ using toluene at 130 °C (bath temperature) in the presence of complex **II** afforded the dehydrogenated quinoline in high yield (88%). The MIC–Ir^III^ complex **II** resulted and active catalyst in thermal dehydrogenation of N-heterocycles at high temperatures. As mentioned earlier, dehydrogenation reactions are endergonic and require high temperatures to proceed. The formation of H_2_ on metal complexes (dihydrogen species) is a high-energy demanding process and the rate-determining step in acceptorless dehydrogenation.^56,57^ In fact, we have previously observed experimentally and confirmed by DFT calculations that the rate-determining step in acceptorless dehydrogenation of alcohols corresponds to the formation of H_2_.^58^ These results suggest a potential operating mode of complex **II** under visible light irradiation consisting in facilitating the formation dihydrogen species. The formation of H_2_ as the rate-determining step implies a resting state intermediate consisting in an iridium-hydride. Experimental evidence of hydride formation in the photodehydrogenation of N-heterocycles was obtained under pseudo-catalytic conditions (13 mol % catalyst loading). A mixture of THQ (5 μL, 0.04 mmol) in the presence of catalyst **II** (5 mg, 0.0053 mmol) in methanol-*d*_4_ was irradiated (blue LEDs) for 3 h. Reaction monitoring by ^1^H NMR confirms the formation of a hydride at −14.23 ppm that is maintained during the reaction, suggesting this intermediate as the resting state (Figure S26). In view of these results, we carried out the synthesis and isolation of the Ir–H complex. The reaction of complex **I** with NaBH_4_ in a mixture of toluene/MeOH afforded the Ir–H complex **IX** as a yellow-orange solid in 83% yield. Ir–H was completely characterized by NMR spectroscopy (showing a characteristic hydride signal at −14.13 ppm) and single crystal X-ray diffraction (Figure 3 and the Supporting Information for details). The absorption spectrum of **IX** was recorded in methanol and compared with the spectra of the related derivatives **I** and **II**. Complex **IX** exhibited a stronger absorption within the blue region, which was confirmed by TD-DFT studies. The photocatalytic properties of the Ir–H complex were evaluated under the same reaction conditions and the results reveal a similar activity than complex **II** (Table S2). These results support Ir–H as active species in the photodehydrogenation of N-heterocycles.

**Figure 4:**
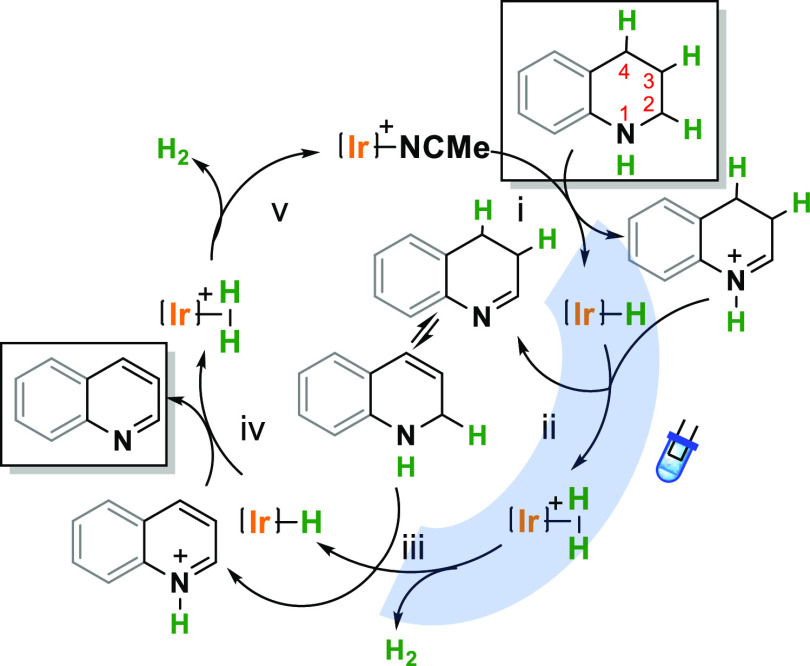
Plausible mechanism in the acceptorless dehydrogenation of N-heterocycles showing the influence of light in hydrogen release.

Based on experimental evidence, precedents in photocatalytic properties of iridium complexes and hydrogenation of N-heterocycles, we proposed a plausible mechanism for the acceptorless photodehydrogenation of N-heterocycles (Figure 4).^59^ An iridium-hydride is formed after acetonitrile dissociation and C2(H) activation, most probably by β-hydride elimination. The origin behind the hydride formation is still an open question and requires further scrutiny. Still, the formation of an Ir–H complex **IX** under the visible light irradiation has been provided, the species isolated, fully characterized, and proven its catalytic activity. THQ is converted into the corresponding 3,4-dihydroquinolin-1-ium (step i). Visible light irradiation induces a singlet to triplet state transition of iridium hydride that increases the hydricity of the Ir–H bond, as it has been established for the Ir–H derivative of complex **V**.^60−62^ This favors the reactivity or the iridium hydride with the low acidic quinolinium intermediate (protonation), forming an iridium–dihydrogen species (step ii). Hydrogen is rapidly released from the iridium–dihydrogen species (step iii) and the process is repeated for the second equivalent of hydrogen (steps iv–v). Dehydrogenation of THQs occurs through a double dehydrogenation. Experimental evidence of dehydrogenation sequence using a model quinoline blocked at C2 position suggests that both dehydrogenations occur at the NH–(C2)H position and not at the remote (C3)H–(C4)H (Figures S27–S29). Tautomerization of 3,4-dihydroquinoline to 1,2-dihydroquinoline precedes the second dehydrogenation that occurs again at the NH–(C2)H position. The release of the second equivalent of H_2_ regenerates the catalyst and provides the final quinoline. Still, further efforts will be necessary to elucidate the exact catalyst–substrate interactions which are currently under investigation in our laboratory. At this stage, a detailed mechanistic study is out of the scope of the present paper. Still, we have provided experimental evidence of the operating mode of the MIC–Ir^III^ complex under light irradiation. This mechanistic proposal also accounts for the hydrogenation of quinoleines using molecular hydrogen which were evaluated using catalyst **II** (Figure S30).

Encouraged by the catalytic performance displayed by MIC–Ir^III^ complex **II** toward the photodehydrogenation of THQs, we evaluated its performance toward the reverse process, that is, the hydrogenation of N-heterocycles using molecular H_2_ as the hydrogen source (Table 3). Hydrogenation of N-heterocycles is exergonic and a well-documented organic transformation where iridium is a preferent catalyst.^63,64^ Based on these precedents, we carried out the iridium hydrogenation (1 mol %) of different quinolines (Qs) using H_2_ (2.5 bar) in toluene at 80 °C for a period of 6 h using a stainless reactor. We were pleased to see that quinoline **1D** was hydrogenated affording the corresponding 1,2,3,4-tetrahydroquinoline **1H** in 99% yield (Figure S31). Similarly, excellent yields were obtained for Qs bearing electron-donating substituent (methyl, methoxy-), except for the 6-Me derivative, which was obtained in a moderate 65% yield. Thus, it can be concluded that the MIC–Ir^III^ complex **II** is also an efficient catalyst for the hydrogenation of quinoline derivatives under mild conditions. As a result, we have proven that a single MIC–Ir^III^ complex **II** is a competent catalyst for the hydrogenation and photodehydrogenation of N-heterocycles.

**Table 3:**
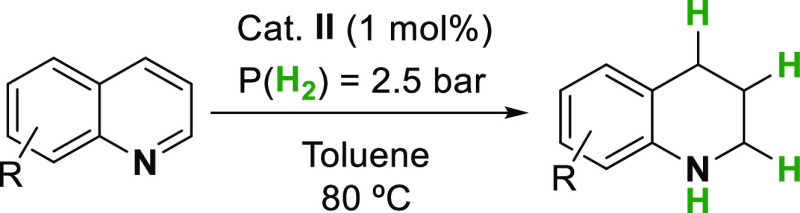

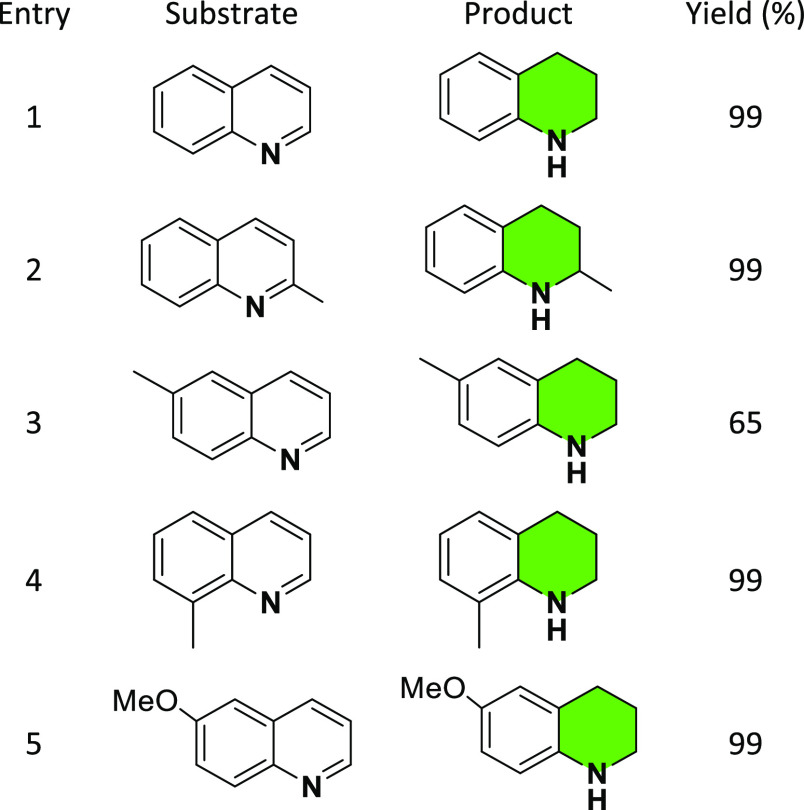
Hydrogenation of N-Heterocycles Using **II**^a^

a Reaction conditions: substrate (0.2 mmol), catalyst (1 mol %), *P*(H_2_) = 2.5 bar, toluene (1 mL) at 80 °C for 6 h. Product formation (yield) obtained by GC/FID using hexadecane as an internal standard.

## Conclusions

In summary, iridium-catalyzed acceptorless dehydrogenation of N-heterocycles can be promoted by visible light. We unveiled a photocatalytic transformation where a single MIC–Ir^III^ is suited to play a dual role, which involves visible-light harvesting, cleavage of N(sp^2^)–H and C(sp^2^)–H bonds with the concomitant formation, and release of H_2_ at room temperature. Experimental evidence supports an iridium-hydride as the resting state whose hydridic properties are enhanced by blue light irradiation, promoting the H_2_ release from N-heterocycles. In addition, this molecular complex efficiently catalyzes the reverse process under mild conditions, which positions itself as a proficient candidate for future hydrogen storage applications.

## Supporting Information Available

Experimental details, synthetic procedures, spectroscopic data, and catalytic studies (PDF)
Crystallographic data for Ir–H (CIF)

## Supplementary Material

cs2c01224_si_001.pdf

cs2c01224_si_002.cif
