# Supplementary material for: Visible-Light-Promoted Iridium(III)-Catalyzed Acceptorless Dehydrogenation of N-Heterocycles at Room Temperature
Source: ACS Catal. 2022 May 10;12(10):6238–45. doi: 10.1021/acscatal.2c01224 (PMC9128065; doi:10.1021/acscatal.2c01224)
Supplement: Supplementary file 1 — cs2c01224_si_001.pdf [file cs2c01224_si_001.pdf]

Visible-Light Promoted Iridium(III) Catalyzed Acceptorless Dehydrogenation of N-heterocycles at Room Temperature

Carmen Mejuto,[a] Laura Ibáñez-Ibáñez, [a] Gregorio Guisado-Barrios[*b] and Jose A. Mata*[*a]*

[a]Institute of Advanced Materials (INAM), Centro de Innovación en Química Avanzada (ORFEO-CINQA), Universitat Jaume I, Avda. Sos Baynat s/n, 12006, Castellón (Spain). Email: jmata@uji.es

[b]Departamento de Química Inorgánica. Instituto de Síntesis Química y Catálisis Homogénea (ISQCH), CSIC-Universidad de Zaragoza, 50009, Zaragoza (Spain).
Email: gguisado@unizar.es

Table of Contents

S1 General procedures 3
S1.1 Reagents and solvents 3
S1.2 Instrumentation 3
S2 Synthesis and characterization of iridium complexes 3
S2.1 Synthesis of triazolium salt **[H_2L]PF_6.** 4
S2.2 Synthesis of iridium complexes 4
S2.3 Characterization NMR spectra 7
S2.4 Single crystal X-ray diffraction data of Iridium hydride 15
S3 Photocatalytic dehydrogenation reactions 17
S3.1 Reaction progress profiles 18
S3.2 ^{1}H NMR spectroscopy analysis in photodehydrogenation reactions 19
S3.3 Photodehydrogenation reactions using Ir-H complex **IX** as catalyst 24
S4 Detection of molecular hydrogen 25
S5 DFT Calculations and Spectroscopic studies 26
S5.1 DFT-generated frontier orbitals and TD-DFT outcome for complex **I** 26
S5.2 DFT-generated frontier orbitals and TD-DFT outcome for complex **II** 28
S5.3 DFT-generated frontier orbitals and TD-DFT outcome of iridium hydride intermediate [**Ir-H**]. 30
S5.4 TD-DFT UV/Vis absorption spectra of MIC-Ir(III) relevant complexes. 31
S5.5 Experimental absorption, excitation, and emission spectra of iridium complexes 32
S6 Thermal dehydrogenation of N-heterocycles 34
S7 Experimental evidence of hydride formation 35
S8 Experimental evidence of dehydrogenation sequence in N-heterocycles induced by visible light 37
S9 Complete mechanistic proposal 38
S10 Hydrogenation of N-heterocycles 39
S11 References 40

S1 General procedures

S1.1 Reagents and solvents

N-heterocycles were purchased from commercial suppliers and used without further purification. Anhydrous solvents were dried using a Solvent Purification System (SPS M BRAUM) or purchased from commercial suppliers degassed and stored over molecular sieves. Solvents used in catalytic experiments were deoxygenated using the freeze-pump-thaw methodology and kept under an atmosphere of nitrogen.

S1.2 Instrumentation

Nuclear magnetic resonance (**NMR**) spectra were recorded on Bruker spectrometers operating at 300 or 400 MHz (^{1}H NMR) and 75 or 100 MHz (^{13}C{^{1}H} NMR), respectively, and referenced to $SiMe_4$ (δ in ppm and J in Hertz). NMR spectra were recorded at room temperature with the appropriate deuterated solvent. Electrospray mass spectra (ESI-MS) were recorded on a Micromass Quatro LC instrument; nitrogen was employed as drying and nebulizing gas. UV-visible absorption spectra were recorded on a Thermo Evolution 600 spectrophotometer. Emission spectra were recorded on a Horiba Fluorolog Jobin Yvon Inc spectrometer using dry and degassed methanol. The photocatalytic reactions were carried out in a homemade photoreactor (see below). The setup has a fan, magnetic stirrer, Schlenk flask connected to a bubbler filled with mineral oil, stirring bar and two 50W blue LEDs (455 nm) lamps (Prilux). All hydrogenation reactions were set up in a 100 mL stainless steel high-pressure reactor. GC analyses were obtained on a Shimadzu GC-2010 apparatus equipped with a FID detector, and using a Teknokroma column (TRB-5MS, 30 m x 0.25 mm x 0.25 μm). Molecular hydrogen was detected using a quadrupole mass spectrometer equipment (Omnistar GSD 320 03 from PFEIFFER VACUUM).

S2 Synthesis and characterization of iridium complexes

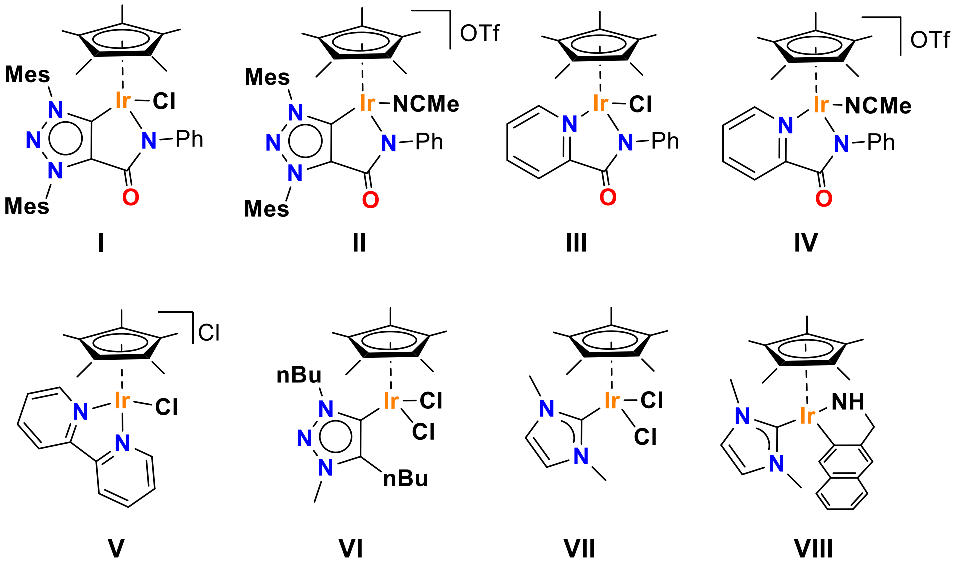

Figure S1 Iridium complexes used in photodehydrogenation of N-heterocycles.

Intermediates used for the synthesis of MIC-ligands (N-phenylpropiolamide **a,** 1,3-bis(2,4,6-trimethylphenyl)triaz-1-ene **b** and *t*-butyl-hypochloride) were prepared following reported methods.[1–3] Pyridine-2-carboxylic acid phenylamide **HL',**[4] and the Ir(III) metal complexes **III**,[5] **IV**,[6] **V**,[7] **VI**,[8] **VII**,[9] and **VIII**,[10] were prepared according to reported procedures.

S2.1 Synthesis of triazolium salt [H_2L]PF_6.

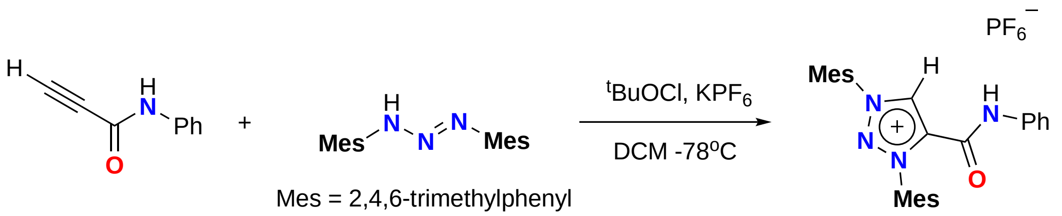

a **b** **[H_2L]PF_6**

Scheme S1 Synthesis of triazolium salt **[H_2L]PF_6**

Synthesis of [[H_2L]PF_6: To a 250 mL Schlenk flask containing **a** N-phenylpropiolamide (1.00 g, 6.8 mmol), **b** 1,3-bis(2,4,6-trimethylphenyl)triaz-1-ene (1.3 eq, 2.52 g, 8.95 mmol) and KPF_6 (1.5 eq, 1.90 g, 10.3 mmol) was added dry dichloromethane (100 mL) under nitrogen and cooled to -78 ^oC. Then, ^{t}BuOCl (0.47 mL, 0.62 mmol) was added dropwise and the solution stirred overnight while allowing warming up to room temperature. The resulting red-brown solution was filtered using a frit funnel to remove a fine precipitate. The volatiles were then removed under reduced pressure to afford a light red powder, which was washed several times with hexane and diethyl ether to afford an ochre powder. 2.04 g, 53% yield. ^1H NMR ($CDCl_3$, 300 MHz, ppm): δ 9.71 (br, 1H, NH), 9.29 (s, 1H, CH_{trz}), 7.74 (d, J_{HH} = 7.9 Hz, 2H, CH_{arom}), 7.30 (t, J_{HH} = 8 Hz, 2H, CH_{arom}), 7.15 (t, J_{HH} = 7.4 Hz, 1H, CH_{arom}), 7.11 (s, 2H, CH_{arom}), 7.10 (s, 2H, CH_{arom}), 2.41 (s, 3H, CH_3), 2.40 (s, 3H, CH_3), 2.18 (s, 6H, CH_3), 2.12 (s, 6H, CH_3); ^{13}C NMR ($CDCl_3$, 75 MHz, ppm) δ 150.5, 143.5, 142.9, 138.2, 136.5, 134.3, 134.2, 132.6, 131.1, 131.0, 130.3, 130.0, 129.2, 121.0, 21.5, 21.4, 17.4, 17.3; HRMS ESI-TOF-MS (positive mode): [M-PF_6]$^+$ 425.2336; calc. 425.2341, ε_r: 1.2 ppm.

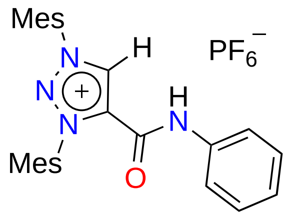

[H_2L]PF_6

S2.2 Synthesis of iridium complexes

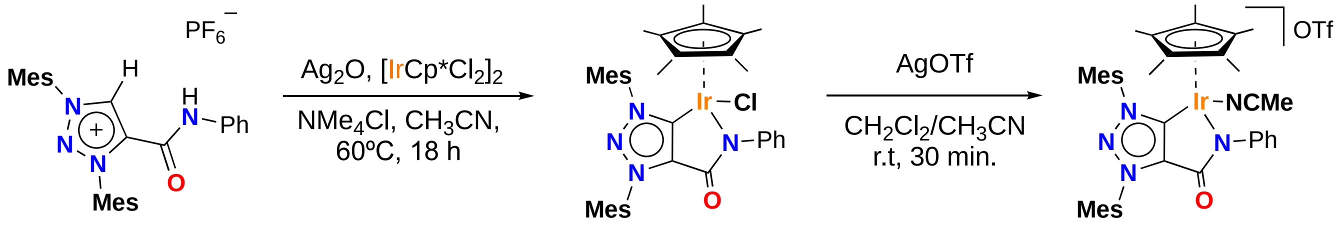

[H_2L]PF_6 **I** **II**

Scheme S2 Synthesis of neutral and cationic MIC-Ir^{III} complexes **I-II**.

Synthesis of [IrCp*Cl(C-N)] I: Under inert atmosphere, 1,3-dimesityl-4-(phenylcarbamoyl)-1H-1,2,3-triazol-3-ium hexafluoro phosphate(V) (0.228 g, 0.4 mmol) **[H_2L]PF_6**, Ag_2O (0.092 g, 0.4 mmol), [Ir{η^5-$C_5(CH_3)_5$}Cl_2]$_2$ (0.159 g, 0.2 mmol) and tetramethyl ammonium chloride (0.131 g, 1.2 mmol) were suspended in dry acetonitrile (7 mL), and stirred for 18 hours at 60 ºC in the absence of light under nitrogen. The reaction mixture was filtered over celite and washed with acetonitrile (2 x 20 mL). The volatiles were removed under reduced pressure. Dichloromethane was added to the crude mixture and filtered over celite again and washed with dichloromethane. The volatiles were removed under reduced pressure to afford the entitle compound as a brown-orange solid, 0.298 g, 95% yield.

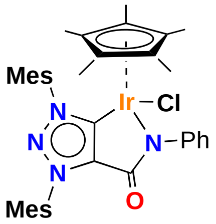

I

^{1}H NMR (400 MHz, CD_2Cl_2, 300 K) δ 7.44 (dd, *J* = 8.4, 1.3 Hz, 2H, CH_{arom}), 7.24(t, J_{HH} = 8.0 Hz, 2H, CH_{arom}), 7.12 (s, 1H, CH_{arom}), 7.08 (s, 1H, CH_{arom}), 7.03 (m, 2H, CH_{arom}), 6.97 (s, 1H), 2.41 (s, 3H, CH_3), 2.40 (s, 3H, CH_3), 2.34 (s, 3H, CH_3), 2.20 (s, 3H, CH_3), 2.12 (s, 6H, CH_3), 1.94 (s, 3H, CH_3), 1.19 (s, 15H, Cp*CH_3); ^{13}C HRMS (100 MHz, CD_2Cl_2, 300 K) δ 163.2, 149.9, 149.7, 148.2, 141.6, 141.3, 137.4, 136.1, 135.9, 135.7, 134.3, 131.7, 130.2, 129.5, 129.4, 129.3, 128.1, 127.9, 124.3, 89.2, 30.3, 21.5, 21.4, 19.1, 18.3, 17.9, 17.7, 9.0. HRMS ESI-TOF-MS (positive mode): [M-Cl]$^+$ 751.2982; calc. 751.2990, ε_r: 1.1 ppm.

Synthesis of [IrCp*($NCCH_3$)(C-N)]OTf, II: Metal complex **II** was prepared by adapting a reported method in literature.[11] To a dichloromethane (15 mL)-acetonitrile (2 mL) solution of **I** (0.1 g, 0.127 mmol) was added AgOTf (0.033 g, 0.127 mmol) at room temperature. After stirring for 30 min, the mixture was filtered through a Celite pad and the filtrate was evaporated under vacuum. The resulting crude mixture was redissolved with 20 mL of dichloromethane and the solution was again filtered through a Celite pad to completely remove AgCl. The solution was evaporated and further dried under vacuum overnight to afford **II** as a brown powder (0.091 g, 76%). ^{1}H NMR (400 MHz, CD_2Cl_2, 300 K) δ 7.26 (t, J_{HH} = 7.7 Hz, 2H, CH_{arom}), 7.12 (s, 2H, CH_{Mes}), 7.07 (t, J_{HH} = 7.4 Hz, 1H, CH_{arom}), 7.01 (s, H, CH_{arom}), 6.93 (d, J_{HH} = 7.8 Hz, 1H CH_{arom}, 2H, CH_{Mes}), 2.40 (s, 3H, CH_3), 2.34 (s, 3H, CH_3), 2.14 (s, 3H, CH_3), 2.12 (s, 6H, CH_3), 1.96 (s, 3H, CH_3), 1.19 (s, 15H, Cp*CH_3); ^{13}C NMR (100 MHz, CD_2Cl_2, 300 K) δ 164.0, 150.4, 149.1, 146.4, 141.8, 141.6, 136.3, 135.5, 135.2, 131.5, 130.1, 130.0, 129.7, 129.4, 128.8, 127.0, 124.3, 90.0, 30.3, 21.5, 21.5, 18.7, 18.0, 17.8, 17.6, 8.62; HRMS ESI-TOF-MS (positive mode): [M]$^+$ 751.2988; calc. 751.2990, ε_r: 0.3 ppm; [M+MeCN]$^+$ 792.3235.

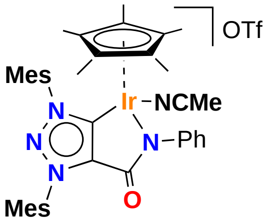

II

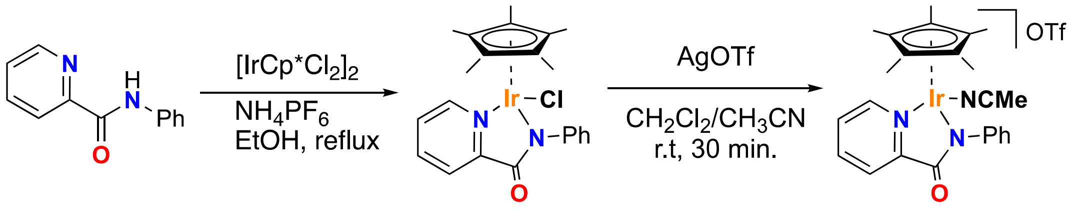

HL’ **III** **IV**

Scheme S3 Synthesis of neutral and cationic Py-Ir^{III} complexes **III-IV**.

Synthesis of [IrCp*Cl(N-N)], III: Complex **III** was prepared by following a reported method in the literature.[5] Pyridine-2-carboxylic acid phenylamide **HL'** (0.2 g, 1.00 mmol) was added to a stirred suspension of [Ir{η^5-$C_5(CH_3)_5$}Cl_2]$_2$ (0.40 g, 0.50 mmol) in ethanol (30 mL) at 95 °C. After 30 min, ammonium hexafluorophosphate (0.40 g, 2.45 mmol) was added and the mixture was stirred at 95 °C for 4 days. The solvent was evaporated, and the residue dissolved in dichloromethane (50 mL), washed with water (2 × 20 mL), brine (20 mL), dried over sodium sulfate, and evaporated to afford the pure compound as an orange solid 0.48 g, 86 % yield. ^{1}H NMR (400 MHz, $CDCl_3$, 300 K) δ 8.56 (ddd, J_{HH} = 5.6, 1.6, 0.8 Hz, 1H, CH_{arom}), 8.14 (d, J_{HH} = 7.8 Hz, 1H, CH_{arom}), 7.90 (m, 1H, CH_{arom}), 7.64 (d, J_{HH} = 7.3 Hz, 2H, CH_{arom}), 7.48 (ddd, J_{HH} = 7.4, 5.5,1.6 Hz, 1H, CH_{arom}), 7.30 (m, 2H, CH_{arom}), 7.08 (ddt, J_{HH} = 8.6, 7.0, 1.2 Hz 1H, CH_{arom}), 1.39 (s, 15H, Cp*CH_3); ^{13}C NMR ($CDCl_3$, 100 MHz, ppm) δ 168.6, 155.6, 149.8, 148.2, 138.6, 128.1, 127.5, 127.0, 126.3, 124.3, 86.7, 8.4; HRMS ESI-TOF-MS (positive mode): [M+H]$^+$ 561.1281; calc. 561.1277, ε_r: 0.7 ppm.

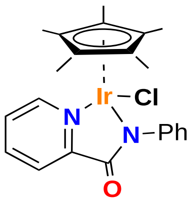

III

Synthesis of [IrCp*(NCCH$_3$)(N-N)]OTf, IV: Metal complex **IV** was prepared by adapting a reported method in literature.[6] To a dichloromethane (15 mL)-acetonitrile (2 mL) solution of **III** (0.207 g, 0.370 mmol) was added AgOTf (0.095 g, 0.370 mmol) at room temperature. After stirring for 30 min, the mixture was filtered through a Celite® pad and the filtrate was evaporated under vacuum. The resulting crude mixture was redissolved with 20 mL of dichloromethane and the solution was again filtered through a Celite pad to completely remove AgCl. The solution was evaporated and further dried under vacuum overnight to afford **IV** as a yellow powder (0.195 g, 74%). ^{1}H NMR (400 MHz, CD_2Cl_2, 400 K) δ 8.68 (d, 1H, J_{HH} = 5.5 Hz, CH_{py}), 7.82 (t, J_{HH} = 7.6 Hz, 1H, CH_{py}), 7.72 (d, J_{HH} = 7.6 Hz, 1H, CH_{py}), 7.51 (t, 1H, J_{HH} = 6.6 Hz, CH_{arom}), 7.23 (dt, J_{HH} = 7.8 Hz, 4H CH_{arom}), 7.10 (t, 1H, J_{HH} = 7.2 Hz CH_{arom}), 1.32 (s, 15H, Cp*CH_3); ^{13}C NMR (100 MHz, CD_2Cl_2, 300 K) δ 169.8, 154.7, 151.0, 148.0, 140.1, 133,3, 128.7, 128.5, 127.0, 126.2, 125.2, 122.7, 119.5, 87.1(Cp*), 8.6 (Cp*); HRMS ESI-TOF-MS (positive mode): [M − OTf – NCCH$_3$]$^+$ 525.1526; calc. 525.1519, ε_r: 1.3 ppm.

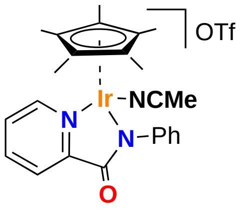

IV

Synthesis of [IrCp*H(C-N)], IX: To an Schlenk containing the iridium complex **I** (1 eq., 0.079 g, 0.1 mmol) dissolved in 3 mL of toluene was added portion wise $NaBH_4$ (10 equiv. 0.038 g, 1 mmol). The reaction mixture was stirred for the period of 1 hour at room temperature. Then 3 mL of methanol were gently added. Once the effervescence has ceased, the volatiles were removed under reduced pressure. Toluene was added to the solid residue and filtered via cannula filtration. The filtrate was dried under vacuum to generate a yellow-orange solid. Yield 83%.

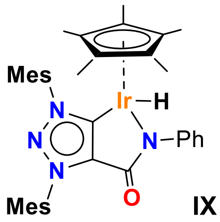

IX

^{1}H NMR (300 MHz, CD_3OD, 300 K) δ 7.28 (t, J = 7.4, 2H, CH_{arom}), 7.15(m, 4H, CH_{arom}), 7.02 (m, 3H, CH_{arom}), 2.41 (s, 3H, CH_3), 2.32 (s, 6H, CH_3), 2.22 (s, 3H, CH_3), 2.17 (s, 3H, CH_3), 1.90 (s, 3H, CH_3),

1.38 (s, 15H, Cp*), -14.13 (s, 1H, Ir-H); ^{13}C NMR (75 MHz, CD_3OD, 300 K) δ 164.3, 152.7, 150.2, 145.6, 142.7, 142.0, 137.2, 136.5, 136.3, 135.9, 135.4, 132.5, 130.7, 130.2, 130.1, 129.0, 128.2, 125.1, 90.8, 90.8, 21.2, 21.2, 19.1, 18.4, 17.5, 17.2, 9.9. HRMS ESI-TOF-MS (positive mode): $[M-H]^+$ 751.2992; calc. 751.2982.

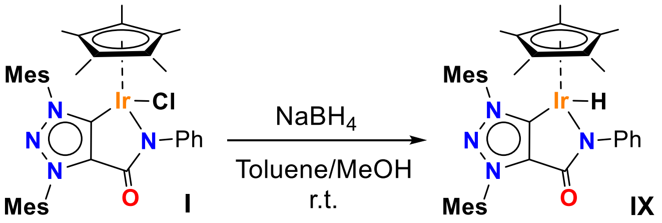

Scheme S4 Synthesis of Iridium hydride complex **IX**.

S2.3 Characterization NMR spectra

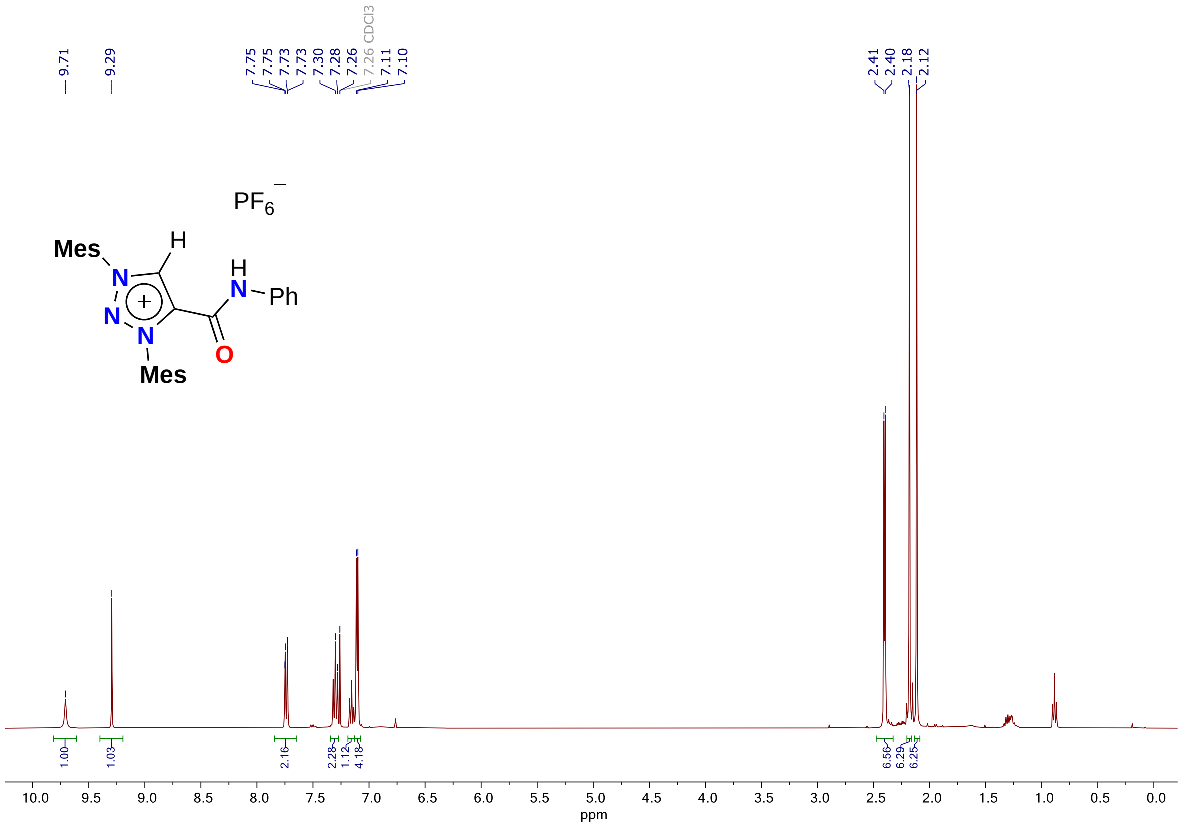

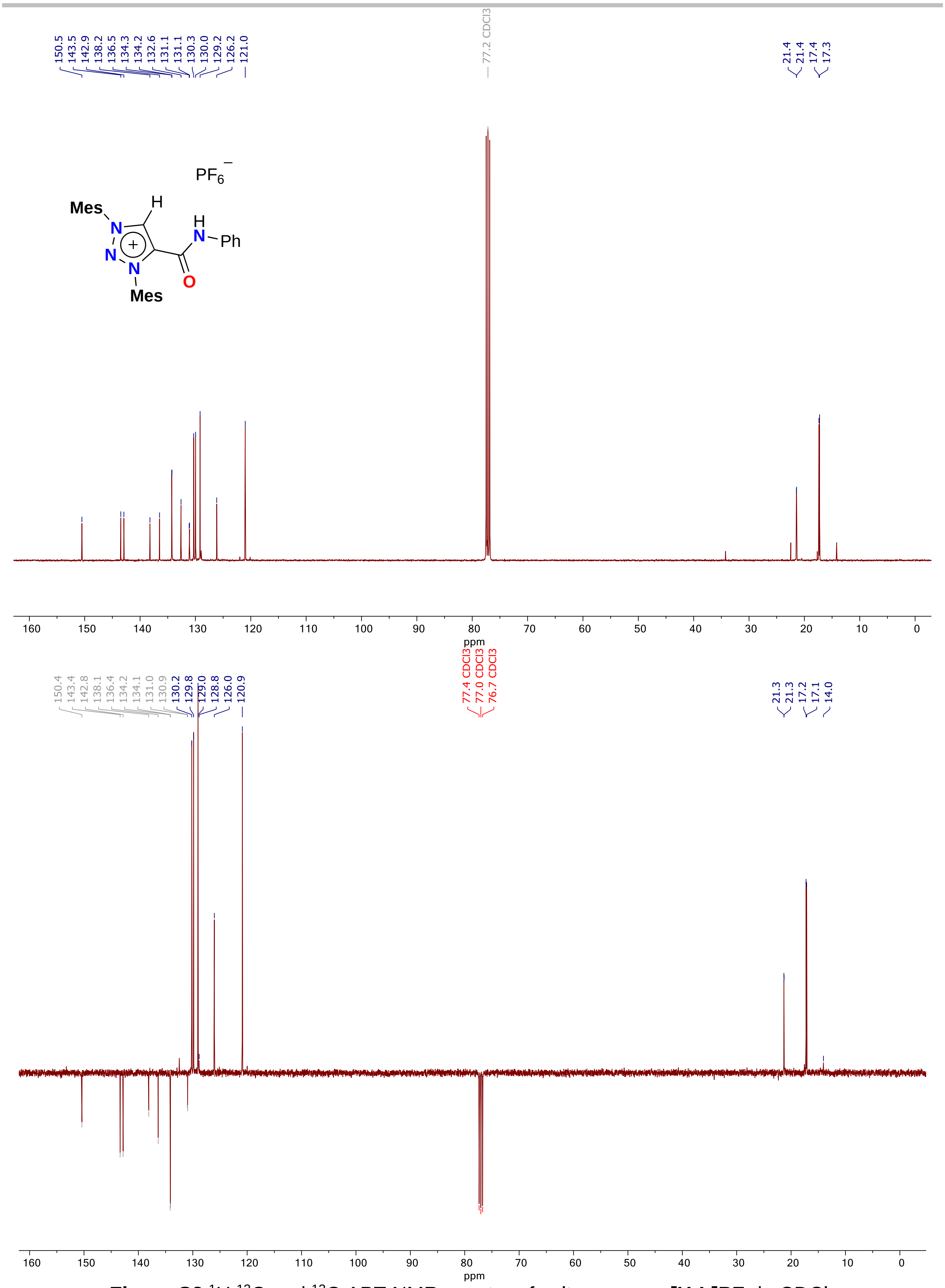

Figure S2 ^{1}H,^{13}C and ^{13}C APT NMR spectra of salt precursor **$[H_2L]PF_6$** in $CDCl_3$

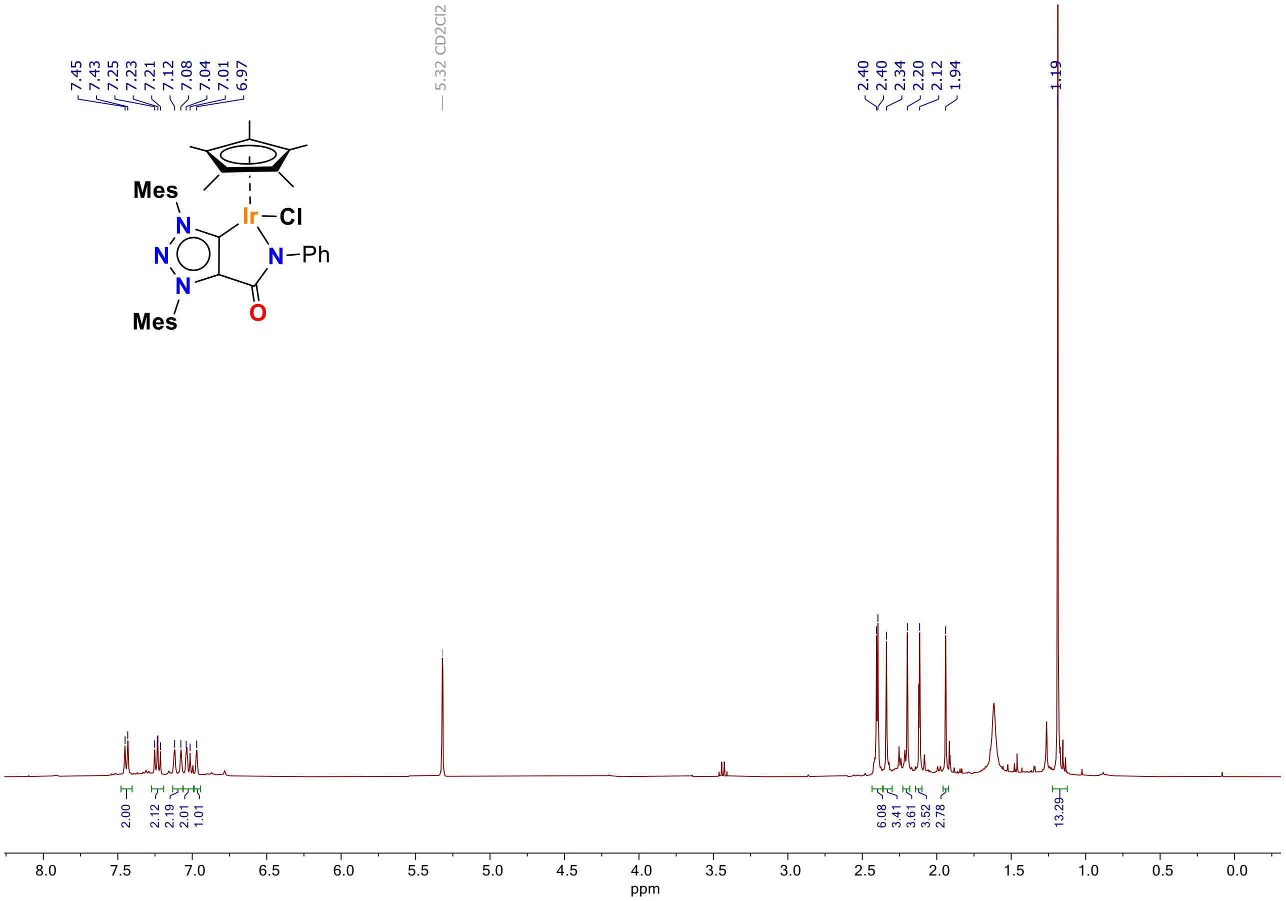

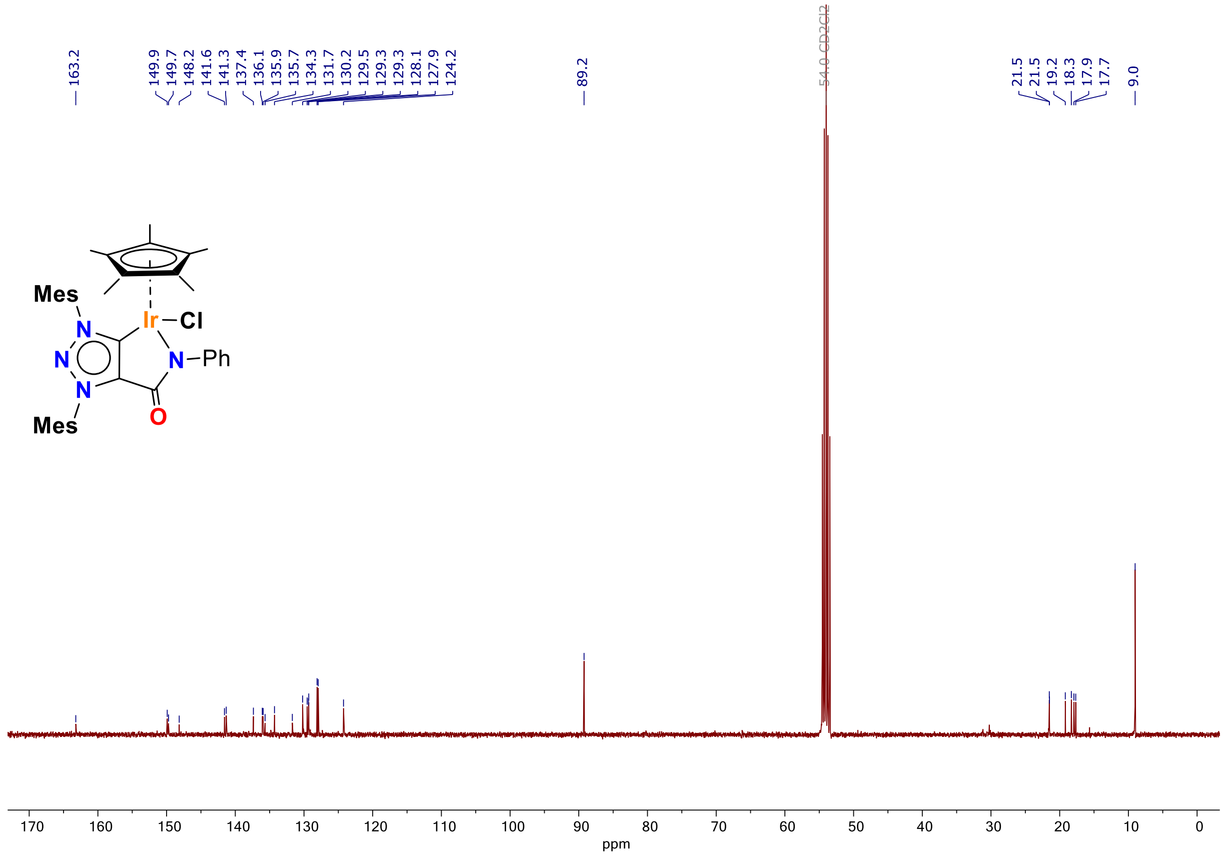

Figure S3 ^{1}H NMR and ^{13}C NMR spectra of complex **I** in CD_2Cl_2

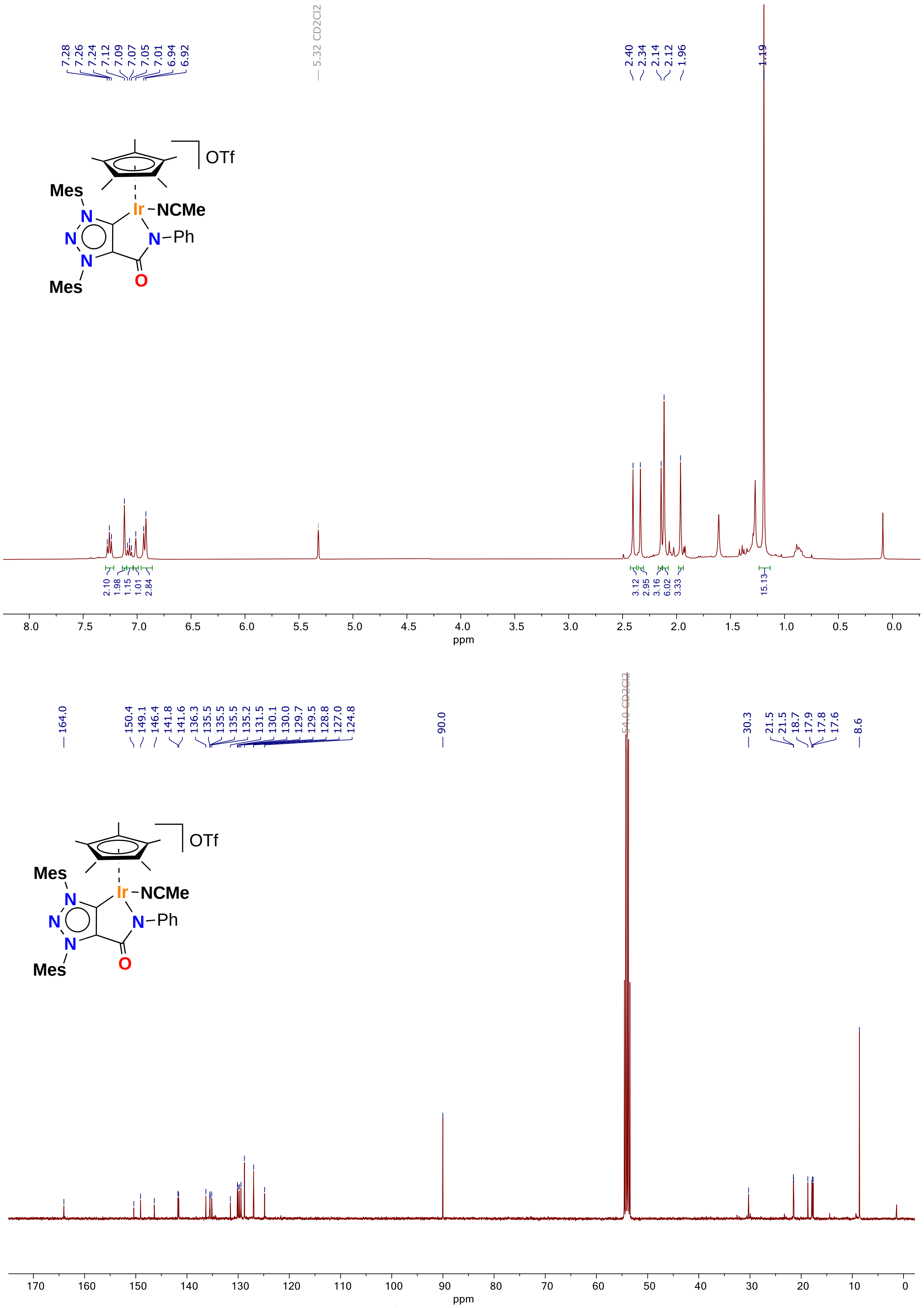

Figure S4 ^{1}H NMR and ^{13}C NMR spectra of complex **II** in CD_2Cl_2

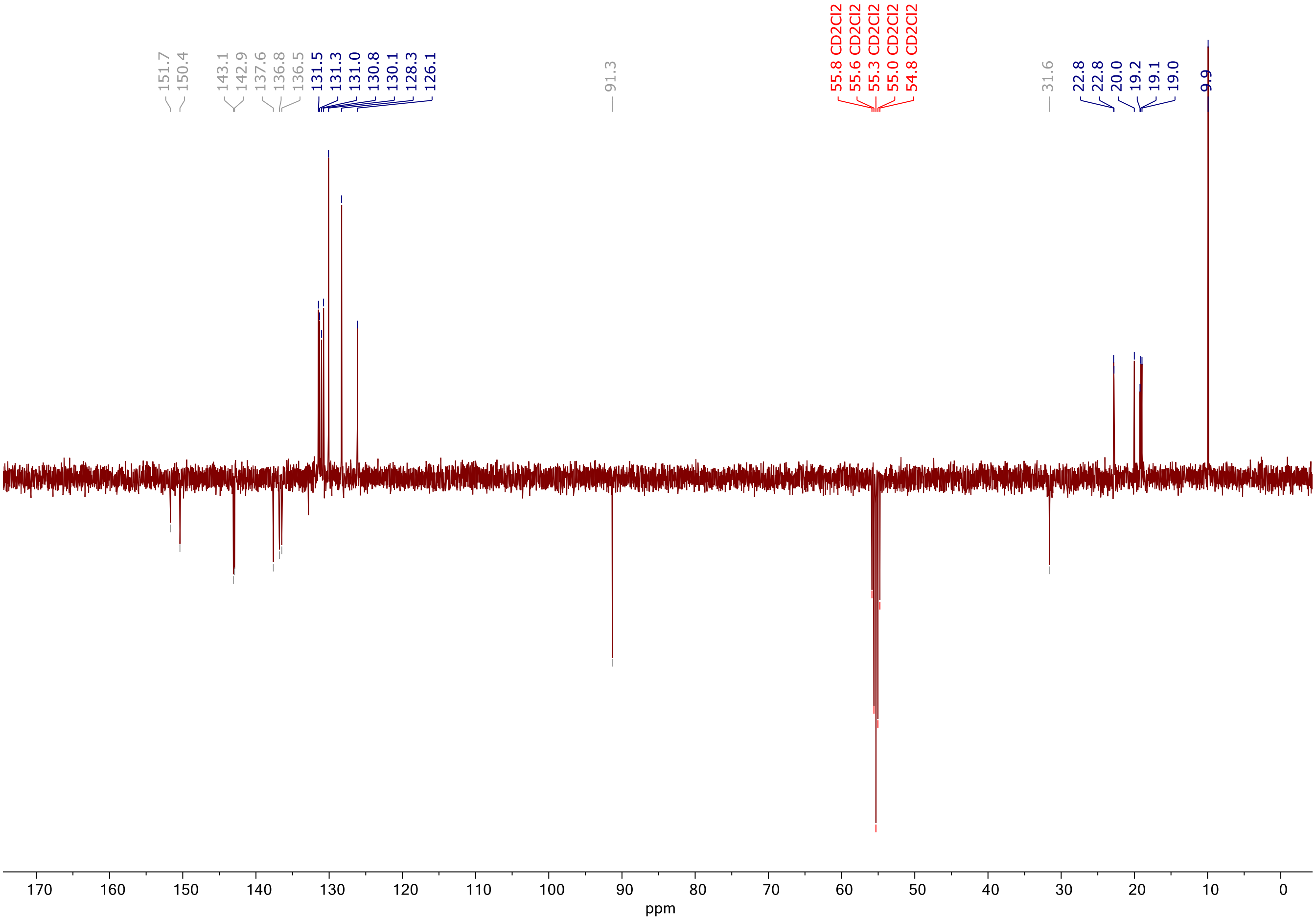

Figure S5 ^{13}C APT NMR spectrum of complex **II** in CD_2Cl_2

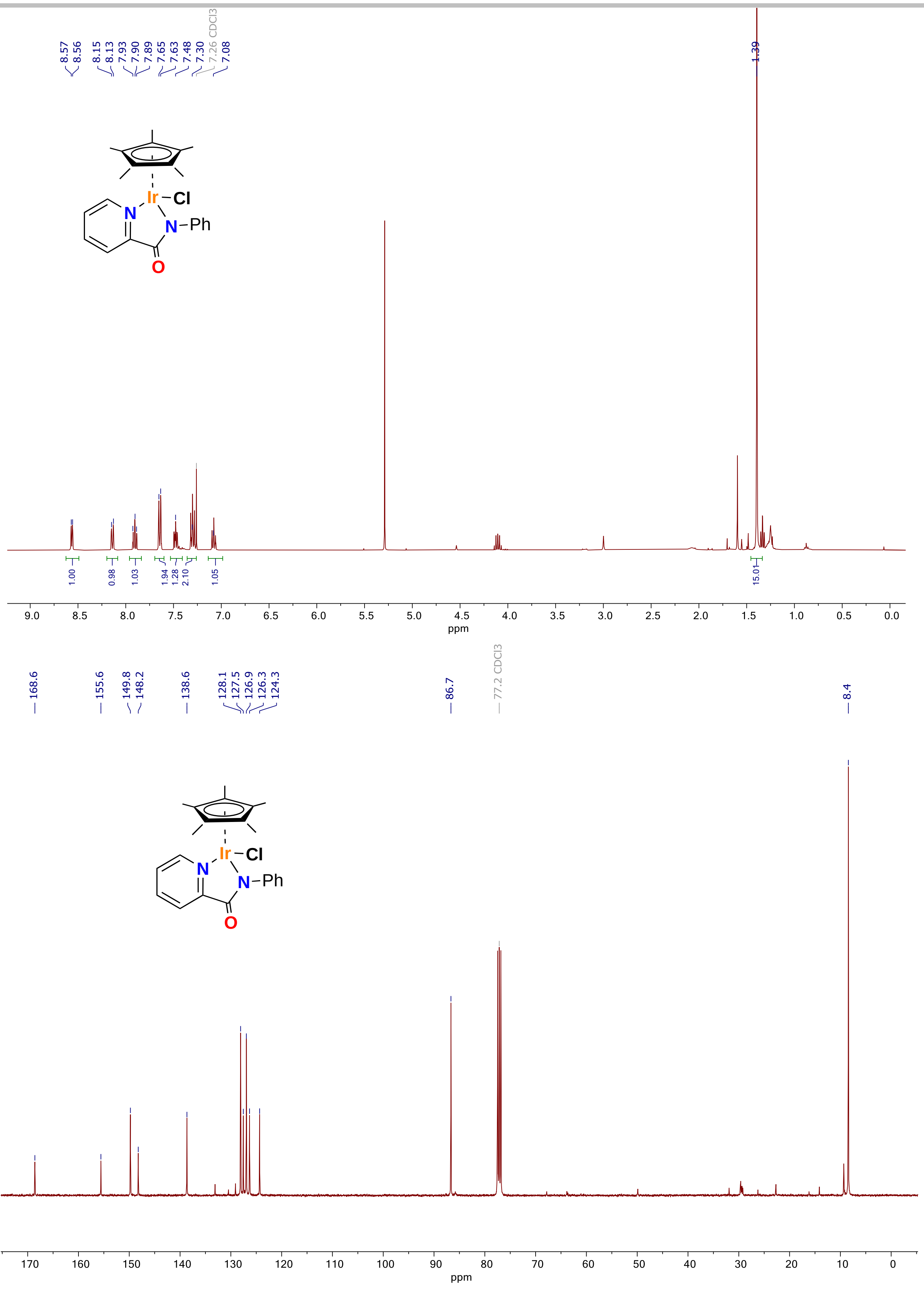

Figure S6 ^{1}H NMR and ^{13}C NMR spectra of complex **III** in $CDCl_3$

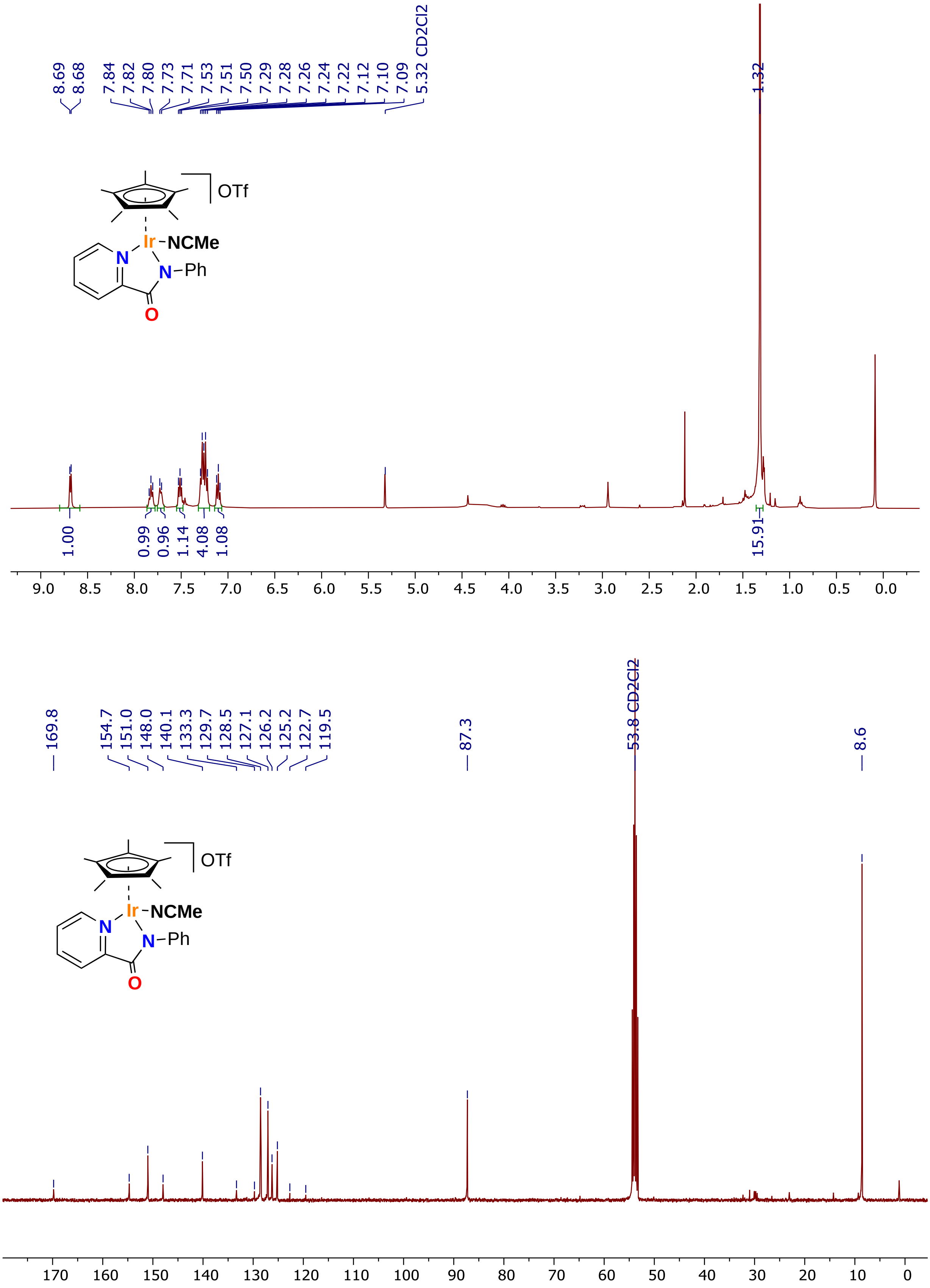

Figure S7 ^{1}H NMR and ^{13}C NMR spectra of complex **IV** in CD_2Cl_2

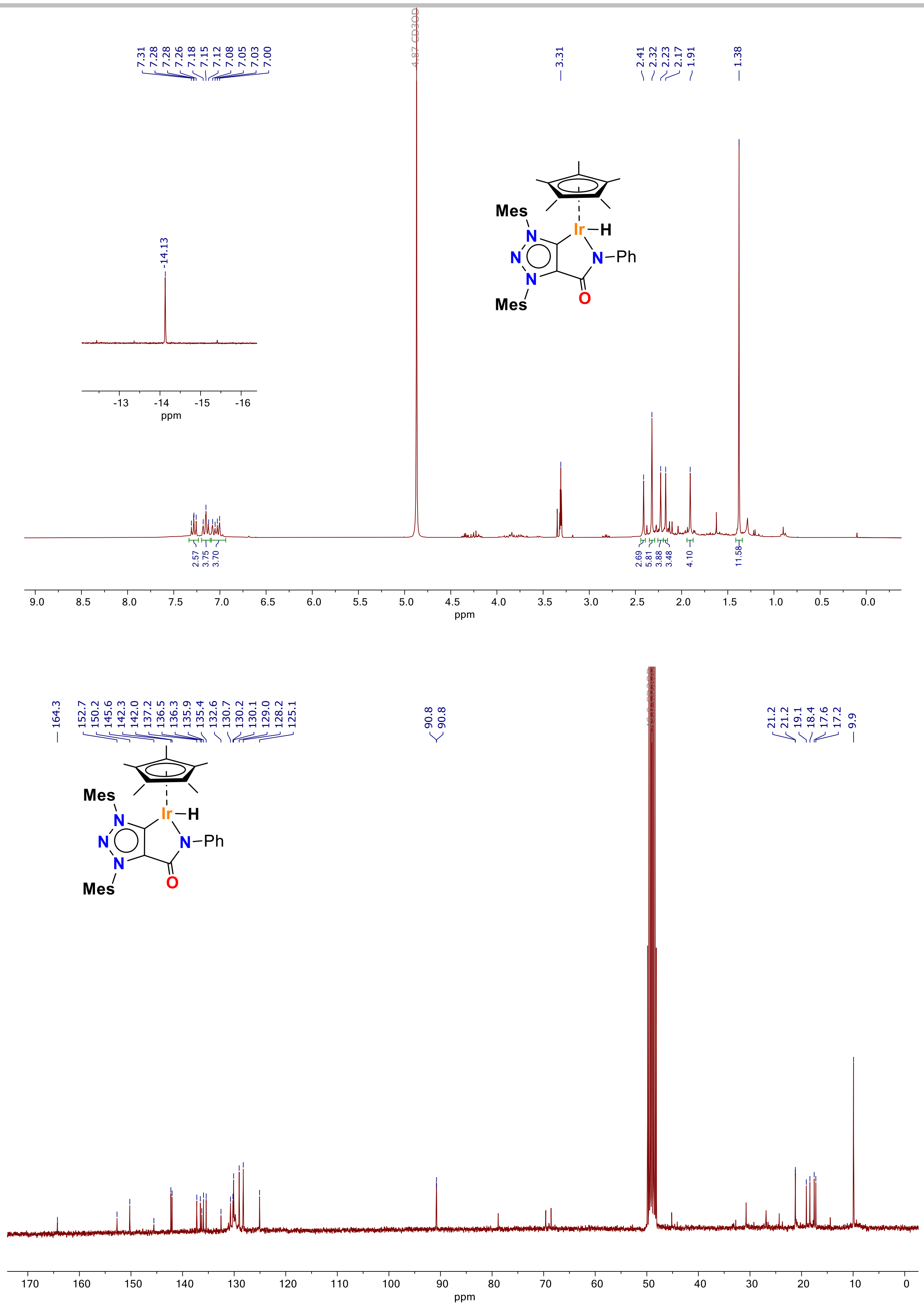

Figure S8 ^{1}H NMR and ^{13}C NMR spectra of complex **IX** in CD_3OD

S2.4 Single crystal X-ray diffraction data of Iridium hydride

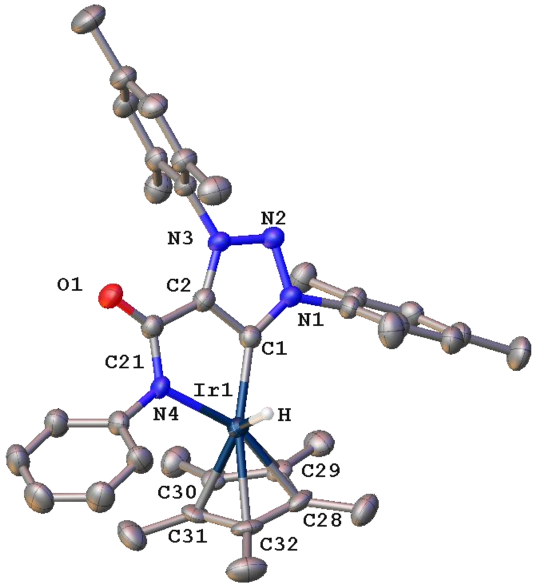

Figure S9. Ortep representation of [Ir-H] complex **IX**. Ellipsoids are at 50% probability level. Hydrogen atoms (except hydride) are omitted for clarity. Selected bond lengths [Å] and angles [º]: Ir(1) - C(1) 2.017(3), Ir(1) - H 1.514, Ir(1) - N(4) 2.144(3), C(1)-Ir(1)- N(4) 76.30, C(1)-Ir(1)- H 82.93, N(4) -Ir(1)- H 60.05

Table S1 Crystal data and structure refinement for Ir-H

Identification code	str2213
CCDC, Deposition Number	2166246
Empirical formula	$C_{38}H_{43}D_4IrN_4O_2$
Formula weight	788.02
Temperature/K	200.00(14)
Crystal system	monoclinic
Space group	$P2_1/c$
a/Å	13.3416(2)
b/Å	17.0392(3)
c/Å	15.4121(3)
α/°	90
β/°	94.170(2)
γ/°	90
Volume/Å^3	3494.36(11)
Z	4
ρ_{calc}g/cm^3	1.498
μ/mm^{-1}	7.686
F(000)	1584.0

Crystal size/mm^3	0.104 × 0.06 × 0.047
Radiation	Cu Kα (λ = 1.54184)
2Θ range for data collection/°	7.746 to 133.2
Index ranges	-15 ≤ h ≤ 14, -20 ≤ k ≤ 20, -18 ≤ l ≤ 18
Reflections collected	30776
Independent reflections	6170 [R_{int} = 0.0359, R_{sigma} = 0.0251]
Data/restraints/parameters	6170/0/423
Goodness-of-fit on F^2	1.072
Final R indexes [I>=2σ (I)]	R_1 = 0.0272, wR_2 = 0.0690
Final R indexes [all data]	R_1 = 0.0336, wR_2 = 0.0714
Largest diff. peak/hole / e Å^{-3}	0.94/-0.67

Experimental

A single crystal of $C_{38}H_{43}D_4IrN_4O_2$ (Ir-H complex) was mounted on a MicroMount® polymer tip (MiteGen) in a random orientation. Data collection was performed on a SuperNova, Dual, Cu at home/near, Atlas diffractometer. The crystal was kept at 200.00(14) K during data collection. Using Olex2 [1], the structure was solved with the SHELXS [2] structure solution program using Direct Methods and refined with the SHELXL [3] refinement package using Least Squares minimisation.

1. Dolomanov, O.V., Bourhis, L.J., Gildea, R.J, Howard, J.A.K. & Puschmann, H. (2009), J. Appl. Cryst. 42, 339-341.
2. Sheldrick, G.M. (2008). Acta Cryst. A64, 112-122.
3. Sheldrick, G.M. (2015). Acta Cryst. C71, 3-8.

Crystal structure determination of Ir-H.

Crystal Data for $C_{38}H_{43}D_4IrN_4O_2$ (*M* =788.02 g/mol): monoclinic, space group $P2_1/c$ (no. 14), *a* = 13.3416(2) Å, *b* = 17.0392(3) Å, *c* = 15.4121(3) Å, *β* = 94.170(2)°, *V* = 3494.36(11) Å^3, *Z* = 4, *T* = 200.00(14) K, μ(Cu Kα) = 7.686 mm^{-1}, *Dcalc* = 1.494 g/cm^3, 30776 reflections measured (7.746° ≤ 2Θ ≤ 133.2°), 6170 unique (R_{int} = 0.0359, R_{sigma}= 0.0251) which were used in all calculations. The final R_1 was 0.0272 (I > 2σ(I)) and wR_2 was 0.0714 (all data).

S3 Photocatalytic dehydrogenation reactions

Photocatalytic dehydrogenation experiments were performed in a 12 mL Schlenk flask connected to a bubbler filled with mineral oil. The bubbler allows the release of hydrogen gas and excludes air from the reaction system. Substrate (0.2 mmol), catalyst (2.0 mol%), 2 x 50 W blue LEDs (455 nm) lamps, dry and deoxygenated methanol (2 mL) and room temperature for the appropriate time. Yield and conversion were determined by GC analysis using hexadecane as internal standard. Isolated yields were determined by solvent evaporation and analysis by ^{1}H NMR spectroscopy.

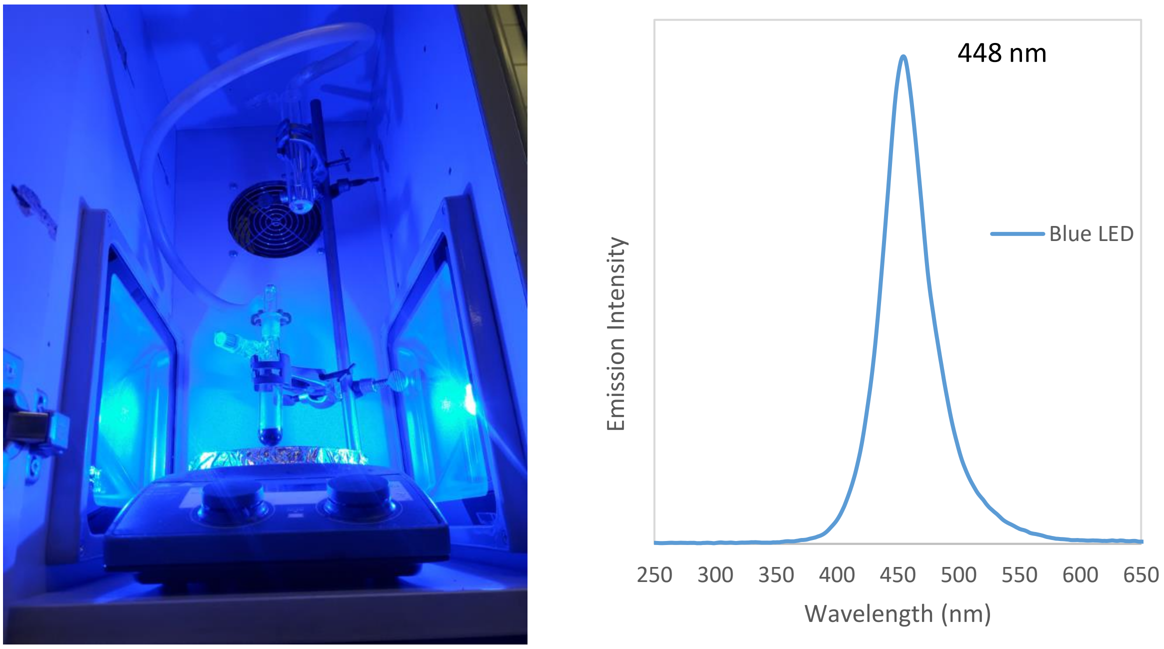

Figure S10 Photoreactor system (left) and emission spectra of Blue LED lamps (right).

S3.1 Reaction progress profiles

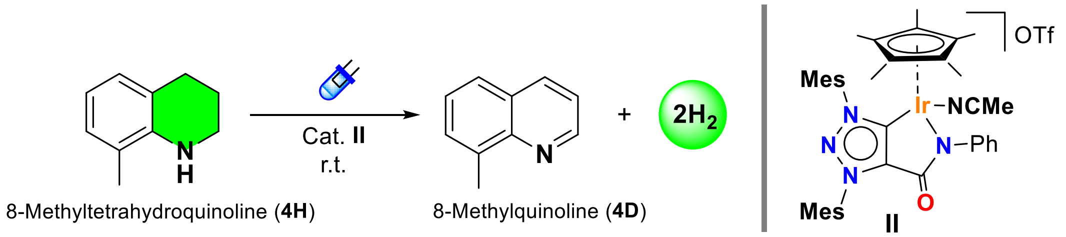

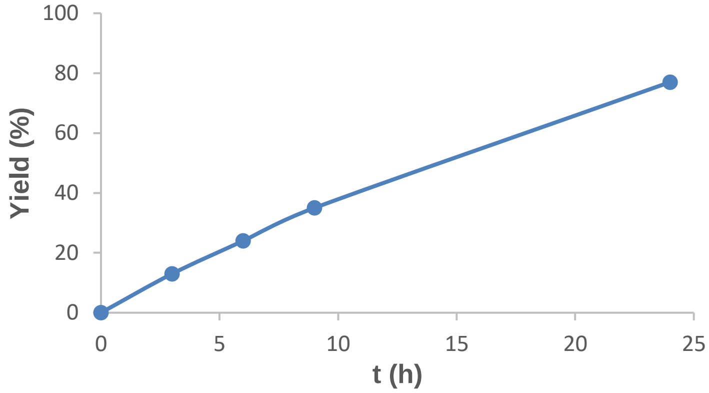

Figure S11 Photocatalytic dehydrogenation of **4H**. Reaction conditions: substrate (0.2 mmol), catalyst **II** (2.0 mol%), methanol (2 mL), blue LEDs (455 nm) at room temperature. Reaction yield (product formation, **4D**) obtained by GC/FID.

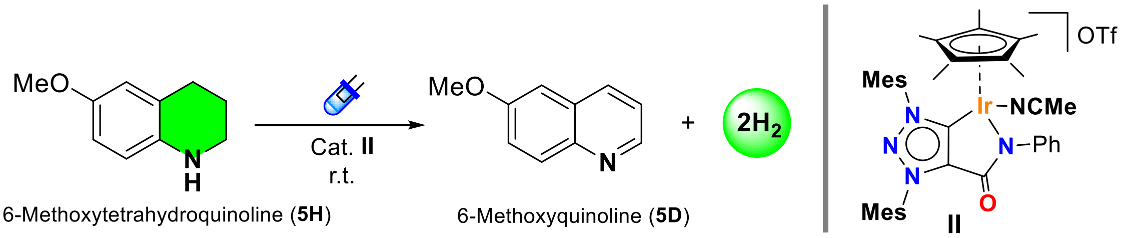

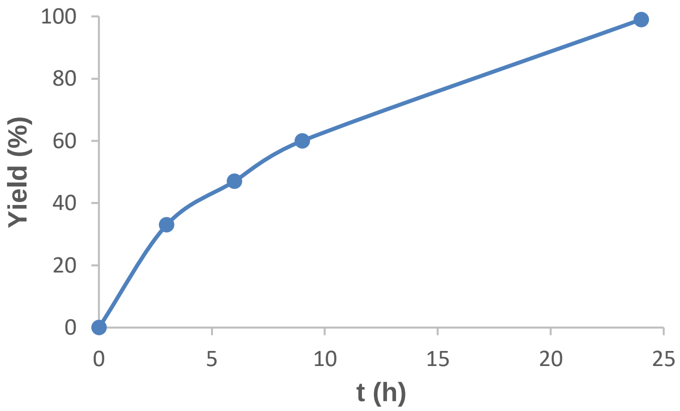

Figure S12 Photocatalytic dehydrogenation of **5H**. Reaction conditions: substrate (0.2 mmol), catalyst **II** (2.0 mol%), methanol (2 mL), blue LEDs (455 nm) at room temperature. Reaction yield (product formation, **5D**) obtained by GC/FID.

S3.2 ^{1}H NMR spectroscopy analysis in photodehydrogenation reactions

Selectivity in photodehydrogenation of N-heterocycles was evaluated by ^{1}H NMR spectroscopy. After 18 h of reaction, the methanol solvent was evaporated, and the remaining mixture was directly analyzed by ^{1}H NMR spectroscopy in $CDCl_3$ without any further purification.

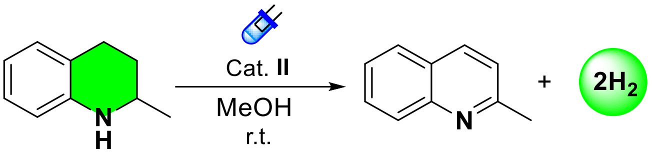

Tetrahydroquinaldine (**2H**) Quinaldine (**2D**)

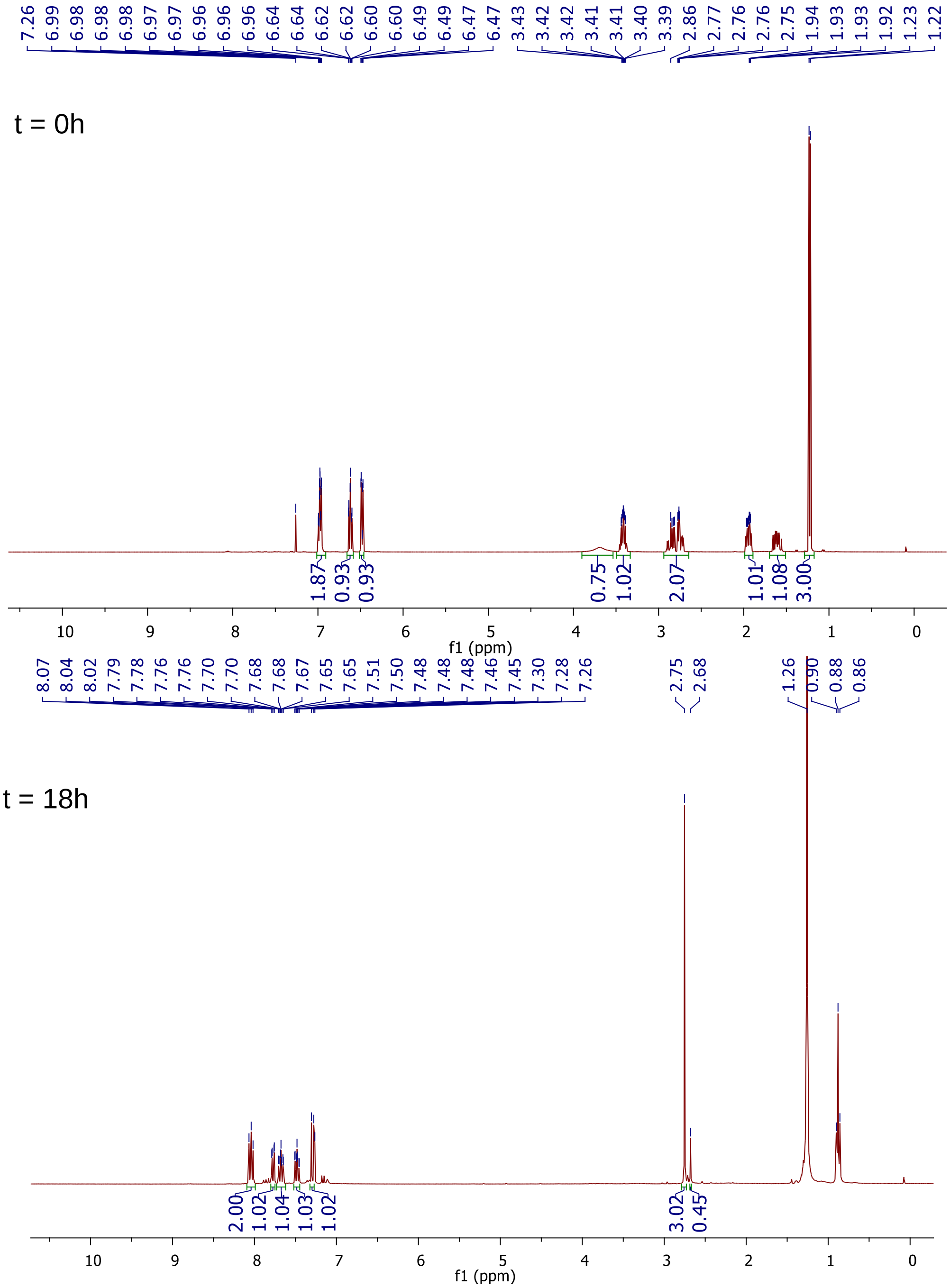

Figure S13 Photodehydrogenation of **2H**. ^{1}H NMR spectrum at t = 0h and t = 18h reaction using hexadecane (signals at 1.26 and 0.88 ppm) as an internal standard. Residual $CDCl_3$ solvent signal at 7.26 ppm.

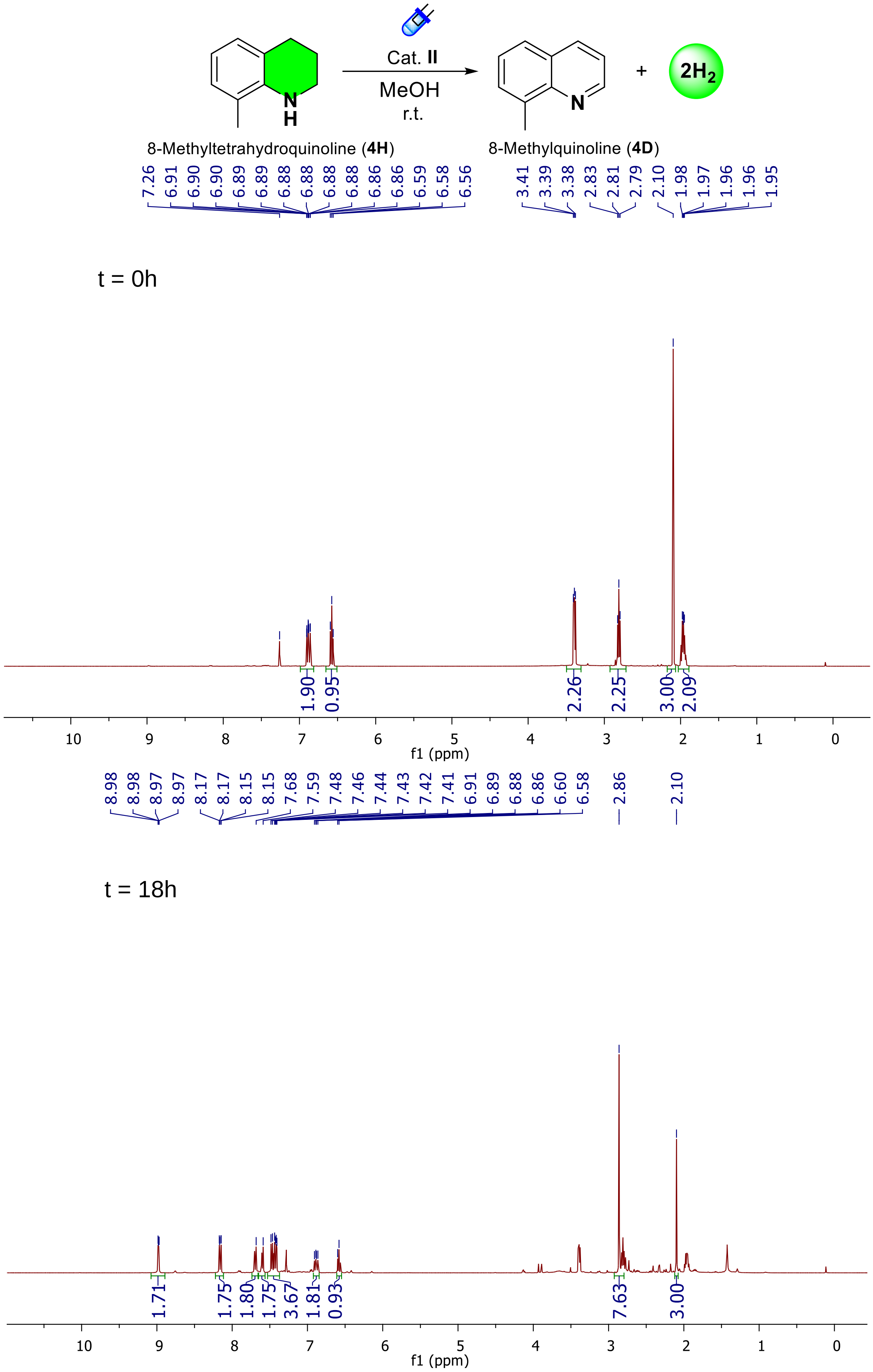

Figure S14 Photodehydrogenation of **4H.** ^{1}H NMR spectrum at t = 0h and t = 18h reaction using hexadecane (signals at 1.26 and 0.88 ppm) as an internal standard. Residual $CDCl_3$ solvent signal at 7.26 ppm.

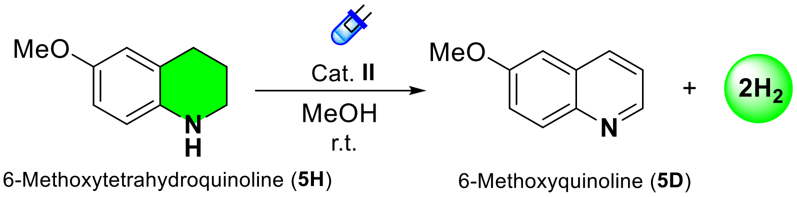

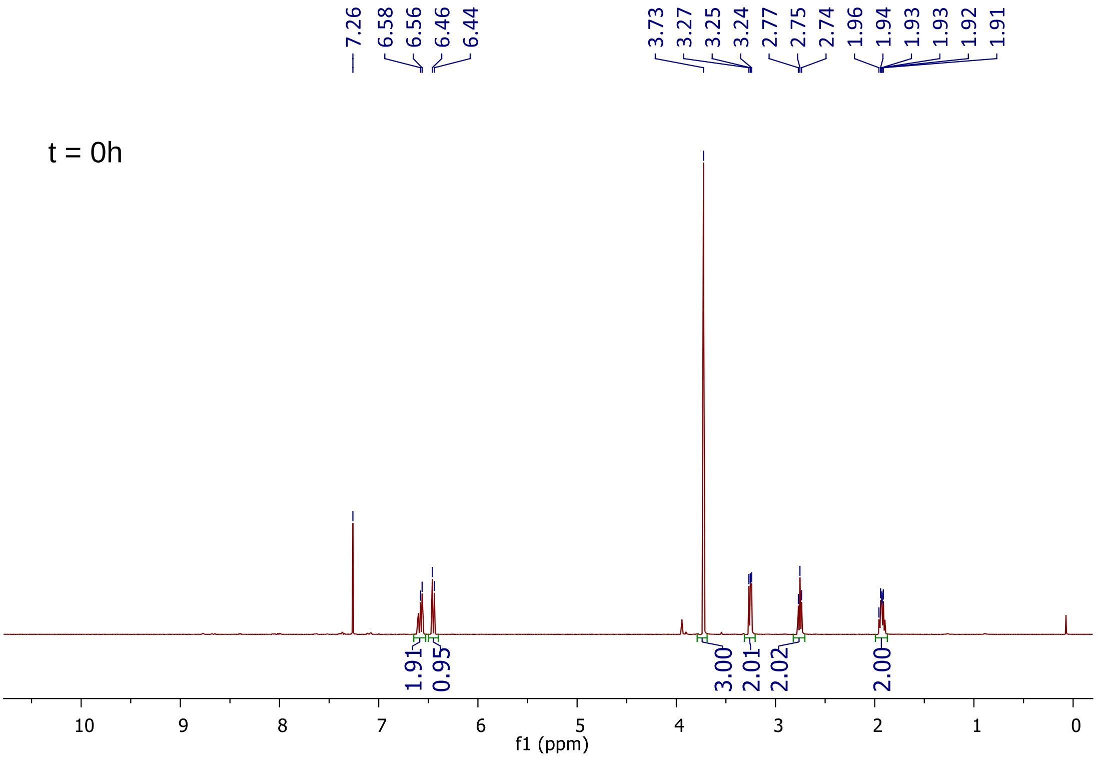

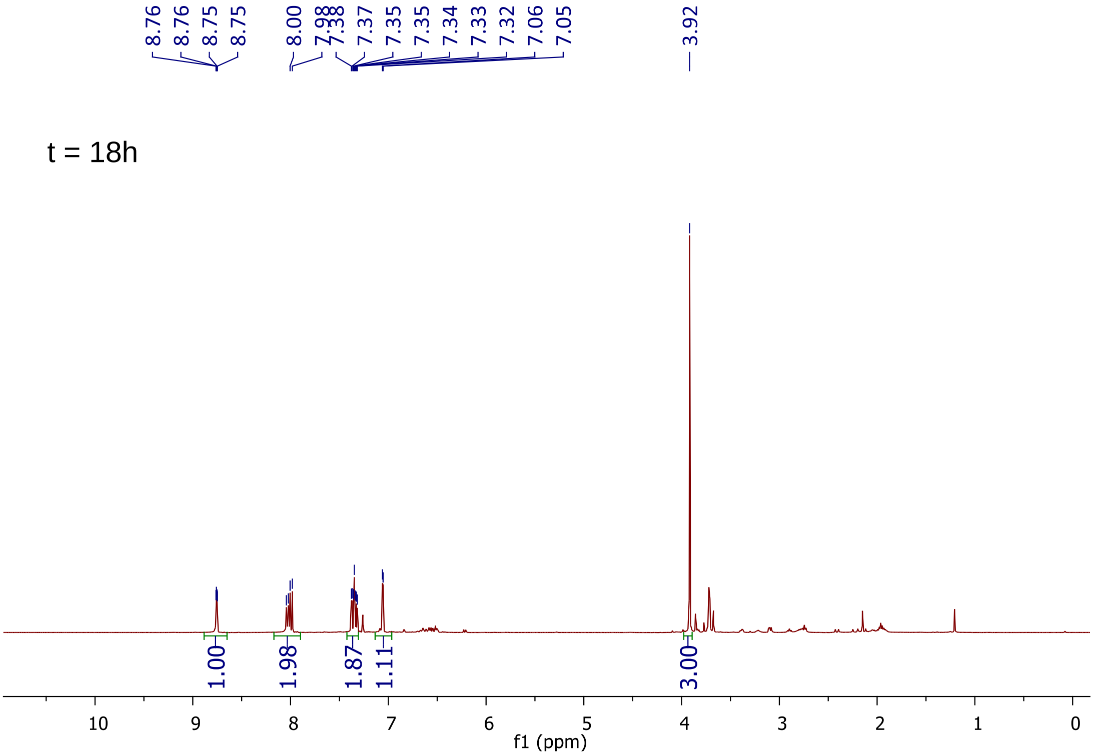

Figure S15 Photodehydrogenation of **5H**. ^{1}H NMR spectrum at t = 0h and t = 18h reaction. Residual $CDCl_3$ solvent signal at 7.26 ppm.

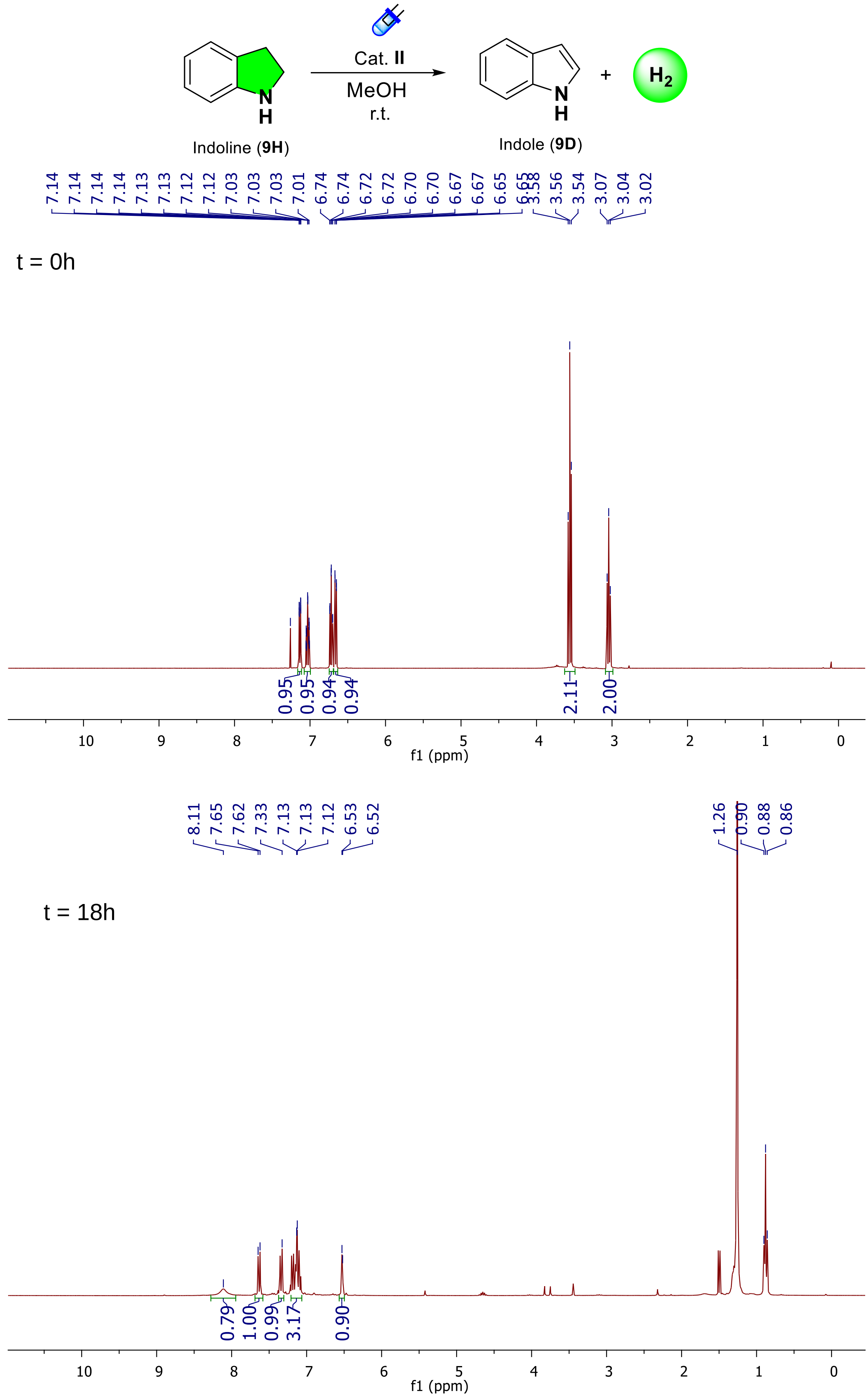

Figure S16 Photodehydrogenation of **9H**. ^{1}H NMR spectrum at t = 0h and t = 18h reaction using hexadecane (signals at 1.26 and 0.88 ppm) as an internal standard. Residual $CDCl_3$ solvent signal at 7.26 ppm.

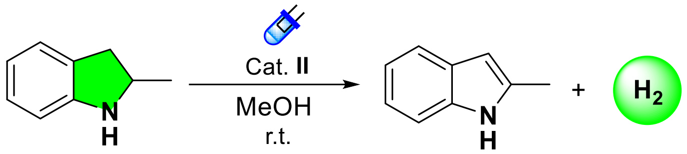

2-Methylindoline (**10H**) 2-Methylindole (**10D**)

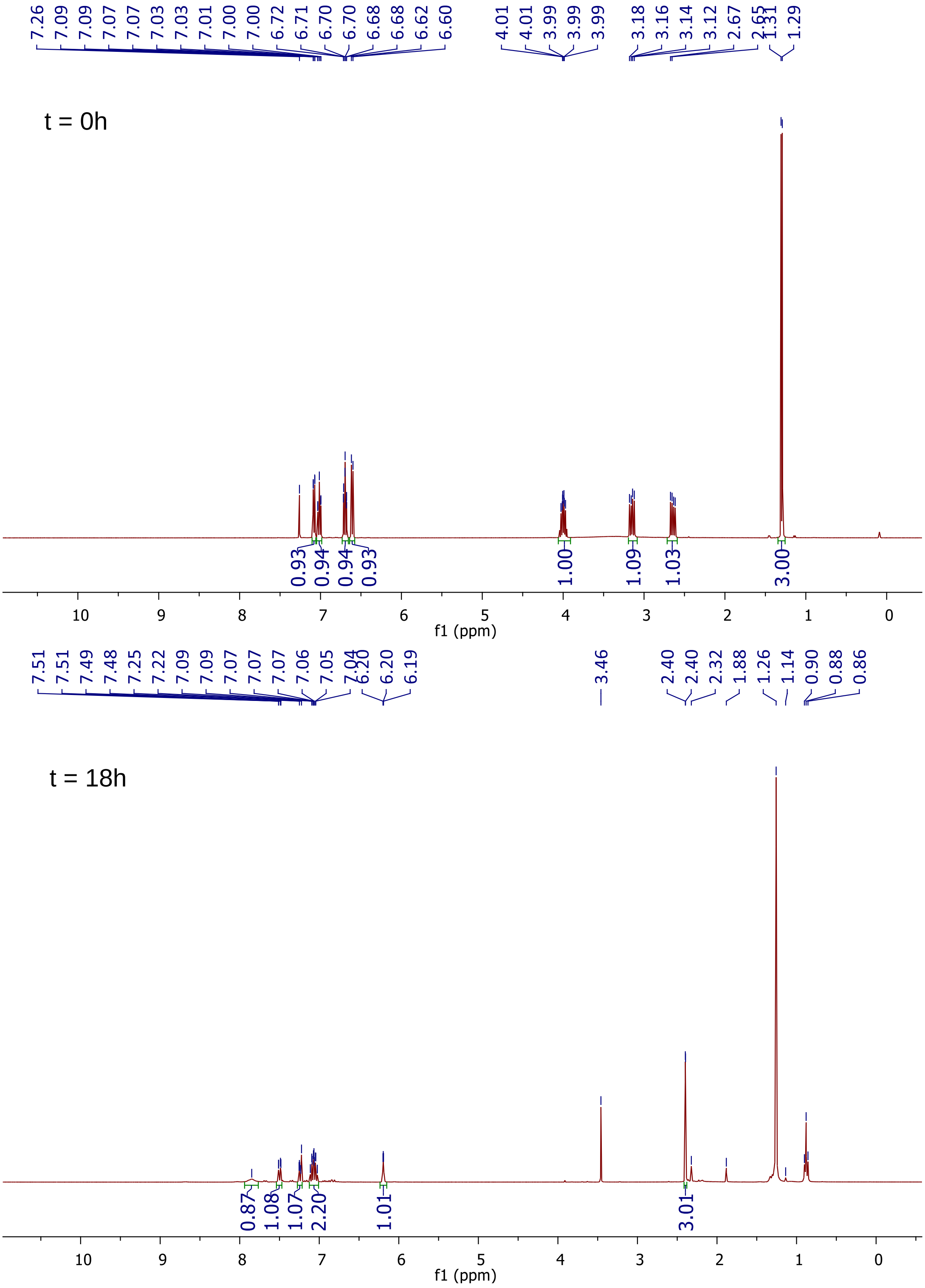

Figure S17 Photodehydrogenation of **10H**. ^{1}H NMR spectrum at t = 0h and t = 18h reaction using hexadecane (signals at 1.26 and 0.88 ppm) as an internal standard. Residual $CDCl_3$ solvent signal at 7.26 ppm and methanol 3.46 ppm.

S3.3 Photodehydrogenation reactions using Ir-H complex **IX** as catalyst

Table S2 Photodehydrogenation of N-heterocycles using Ir-H complex **IX**.

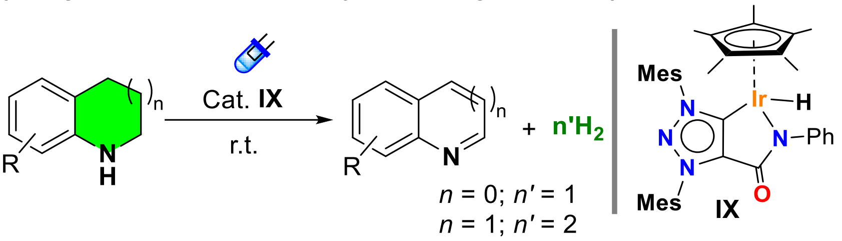

Entry	Subs.	Prod.	T (°C)	Time (h)	Yield[a] (%)
1	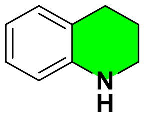	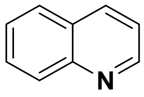	r.t.	18	84
2	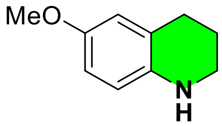	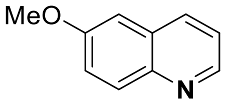	r.t.	18	91
3	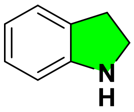	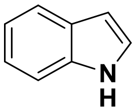	r.t.	18	83
4	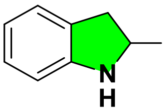	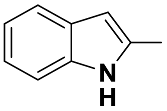	r.t.	18	73
5[b]	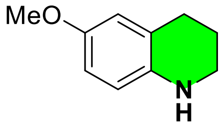	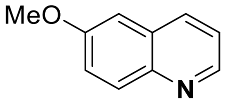	r.t.	18	8

Reaction conditions: substrate (0.2 mmol), Ir-H catalysts (2.0 mol%), methanol (2 mL) and blue LEDs (455 nm). a) Yield obtained by GC/FID using hexadecane as an internal standard. b) Reaction under dark conditions.

S4 Detection of molecular hydrogen

In a 12 mL Schlenk flask the stopcock valve was connected to a bubbler filled with mineral oil to exclude air from the reaction system and allowing the release of hydrogen gas and the flask was capped with a septum rubber. This septum rubber was used to introduce a syringe to capture the generated gas. Substrate (0.2 mmol), catalyst (2.0 mol%), blue LEDs (455 nm), dry/deoxygenated methanol (2 mL) and room temperature. After 5h reaction, the gas evolved was collected with a syringe and injected in a quadrupole mass spectrometer with a TCI detector (Omnistar GSD 320 03 from PFEIFFER VACUUM) confirming the presence of molecular hydrogen. The same experiment was repeated in the absence of catalyst as control.

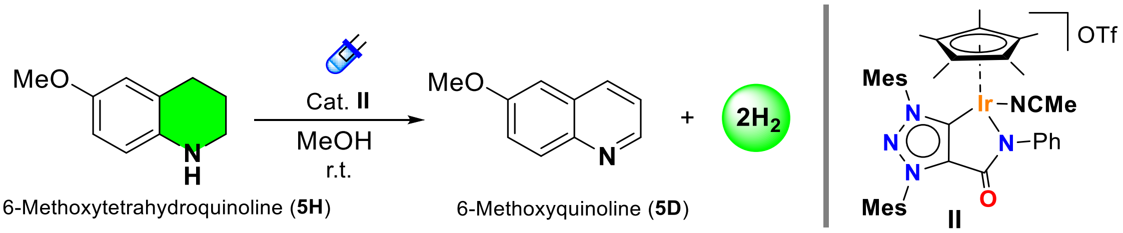

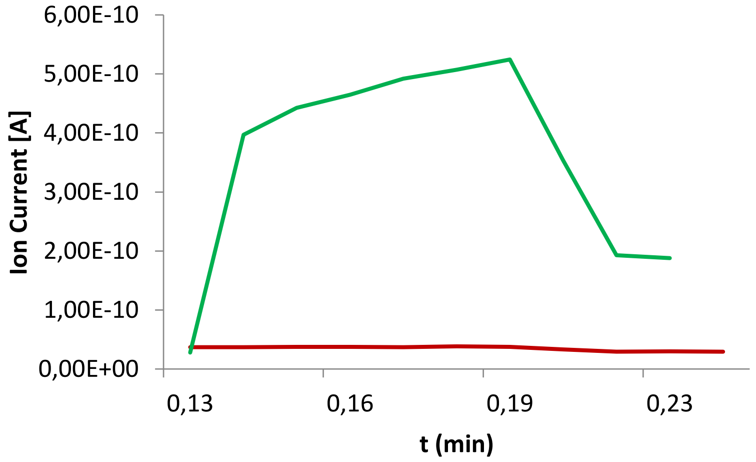

Figure S18 Detection of molecular hydrogen in the photodehydrogenation of **5H** using catalyst **II** (green line) and control experiment without catalyst (red line).

S5 DFT Calculations and Spectroscopic studies

Geometry optimizations, single-point calculations, frequency calculations and time-dependent calculations (TD-DFT) were performed using Gaussian 09 suite of programs with the PBE1PBE functional and the LanL2DZ basis for Ir and 6-31G(d) for (C H N O).[12] Frequency calculations were performed on optimized geometries to ensure true minima.

S5.1 DFT-generated frontier orbitals and TD-DFT outcome for complex **I**

Table S3 DFT generated frontier orbitals (MO = 165, MO = 166, MO = 167, MO = 168, MO = 169, MO = 170) of iridium complex **I**.

MO	Occupancy	Energy (eV)
170	□	-0.54
169	□	-0.98
168	□	-1.47
167	↑↓	-5.56
166	↑↓	-6.07
165	↑↓	-6.54

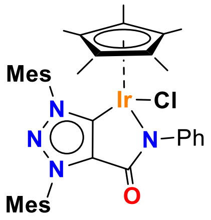

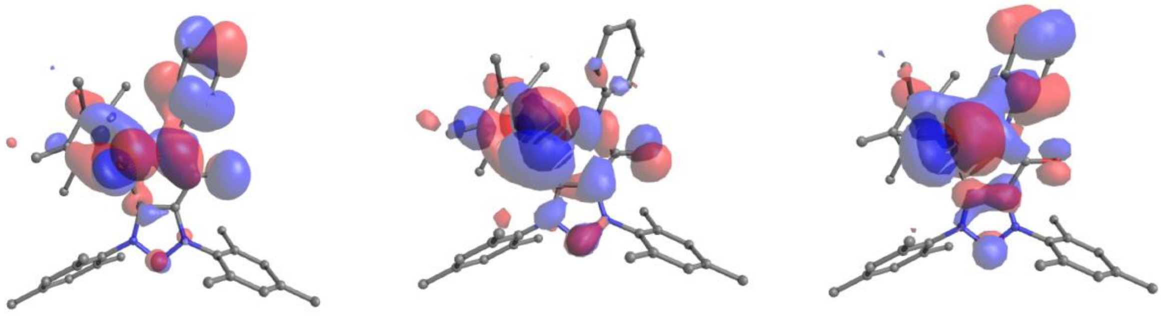

HOMO (MO = 167) HOMO-1 (MO = 166) HOMO-2 (MO = 165)

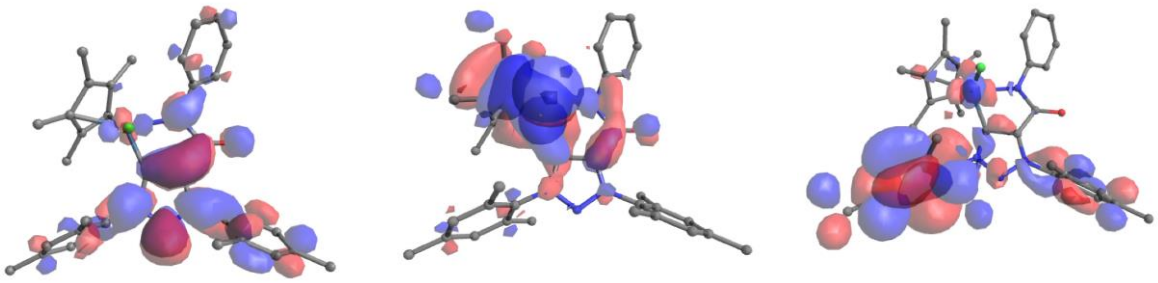

LUMO (MO = 168) LUMO+1 (MO = 169) LUMO+2 (MO = 170)

Figure S19 Electronic density distribution of selected DFT-generated frontier orbitals of complex **I** (H atoms are omitted for clarity).

Table S4 DFT outcome: Orbital composition of the calculated singlet excitation (first four) of iridium complex **I**. Relevant transitions are marked in bold.

**Excited state 1**				
409 nm (*f* =0.0128)	**167 (HOMO)**	⟶	**169 (LUMO+1)**	**(0.83)**
	167 (HOMO)	⟶	168 (LUMO)	(0.02)
**Excited state 2**				
379 nm (*f* =0.0560)	**167 (HOMO)**	⟶	**168 (LUMO)**	**(0.94)**
	167 (HOMO)	⟶	169 (LUMO+1)	(0.02)
**Excited state 3**				
355 nm (*f* =0.0153)	**166 (HOMO-1)**	⟶	**169 (LUMO+1)**	**(0.86)**
	165 (HOMO-2)	⟶	169 (LUMO+1)	(0.02)
**Excited state 4**				
333 nm (*f* =0.0208)	**166 (HOMO-1)**	⟶	**168 (LUMO)**	**(0.94)**

S5.2 DFT-generated frontier orbitals and TD-DFT outcome for complex **II**

Table S5 DFT-generated frontier orbitals (MO = 169, MO = 168, MO = 169, MO = 170, MO = 171, MO = 172, MO 173) of iridium complex **II**.

MO	Occupancy	Energy (eV)
173	□	-2.892
172	□	-3.036
171	□	-3.423
170	□	-3.841
169	↑↓	-7.882
168	↑↓	-8.755
167	↑↓	-8.946

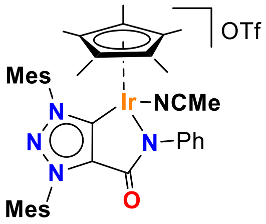

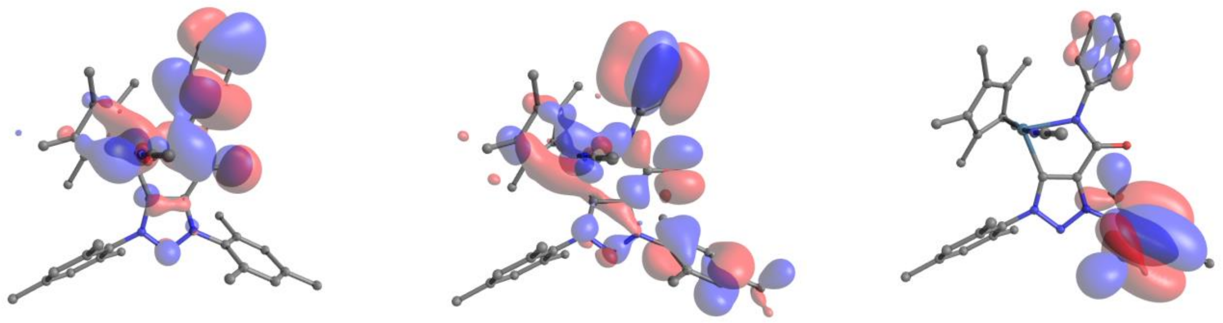

HOMO (MO = 169) HOMO-1 (MO = 168) HOMO-2 (MO = 167)

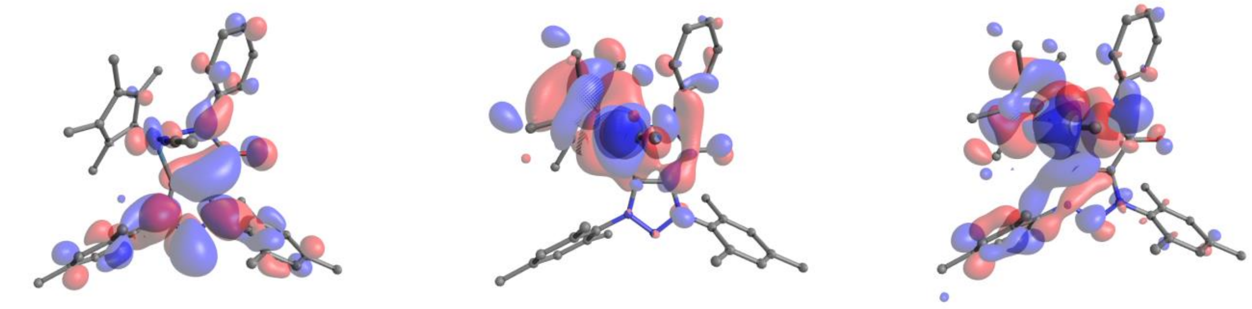

LUMO (MO = 170) LUMO+1 (MO = 171) LUMO+3 (MO = 173)

Figure S20 Electronic density distribution of selected DFT-generated frontier orbitals of complex **II** (H atoms are omitted for clarity).

Table S6 TD-DFT outcome: Orbital compositions of the calculated singlet excitations (first four) of iridium complex **II**. Relevant transitions are marked in bold.

**Excited State 1**				
366 nm (*f* =0.0593)	**169 (HOMO)**	⟶	**171 (LUMO+1)**	**(0.51)**
	169 (HOMO)	⟶	170 (LUMO)	(0.39)
**Excited State 2**				
358 nm (*f* =0.0678)	169 (HOMO)	⟶	171 (LUMO+1)	(0.35)
	**169 (HOMO)**	⟶	**170 (LUMO)**	**(0.59)**
**Excited State 3**				
311 nm (*f* =0.0235)	169 (HOMO)	⟶	173 (LUMO+3)	(0.26)
	168 (HOMO-1)	⟶	171 (LUMO+1)	(0.10)
	**169 (HOMO-1)**	⟶	170 **(LUMO)**	(0.31)
**Excited State 4**				
307 nm (*f* =0.0036)	169 (HOMO)	⟶	173 (LUMO+3)	(0.26)
	**168 (HOMO-1)**	⟶	**170 (LUMO)**	(0.31)

S5.3 DFT-generated frontier orbitals and TD-DFT outcome of iridium hydride intermediate [**Ir-H**].

Table S7 DFT generated frontier orbitals (MO = 157, MO = 158, MO = 159, MO = 160, MO = 161, MO = 162) of **[Ir-H]**.

MO	Occupancy	Energy (eV)
163	□	-0.22
162	□	-0.49
161	□	-1.40
159	↑↓	-5.20
158	↑↓	-5.68
157	↑↓	-6.46

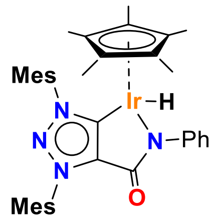

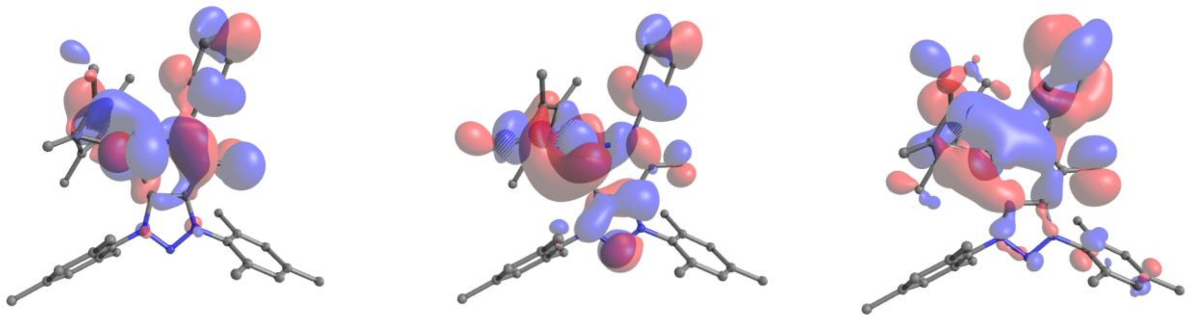

HOMO (MO = 159) HOMO-1 (MO = 158) HOMO-2 (MO=157)

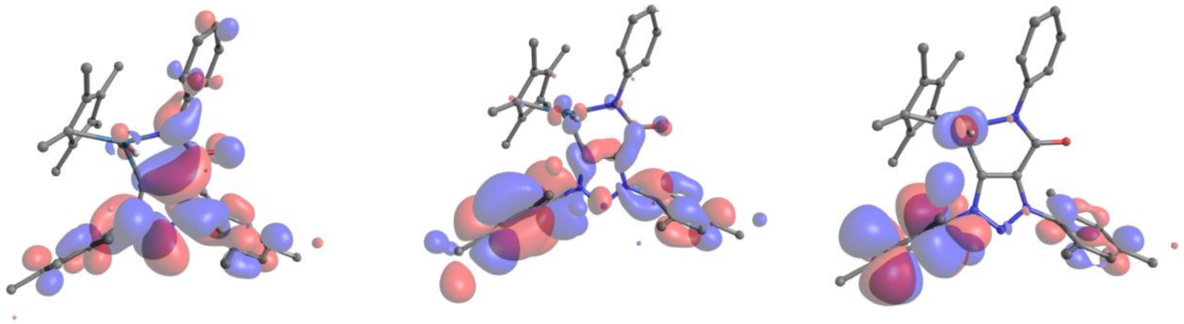

LUMO (MO = 160) LUMO+1 (MO = 161) LUMO+2 (MO=162)

Figure S21 Electronic density distribution of selected DFT-generated frontier orbitals of **[Ir-H]** (H atoms are omitted for clarity).

Table S8 DFT outcome: Orbital composition of the calculated singlet excitation (first four) of **[Ir-H]**. Relevant transitions are marked in bold.

**Excited state 1**				
417 nm (*f* =0.0174)	**159 (HOMO)**	⟶	**160 (LUMO)**	**(0.99)**
**Excited state 2**				
355 nm (*f* =0.11)	**158 (HOMO-1)**	⟶	**160 (LUMO)**	**(0.95)**
	156 (HOMO-3)	⟶	160 (LUMO)	(0.02)
**Excited state 3**				
341 nm (*f* =0.0084)	159 (HOMO)	⟶	161 (LUMO+1)	(0.17)
	159 (HOMO)	⟶	163 (LUMO+3)	(0.26)
	**159 (HOMO)**	⟶	**165 (LUMO+5)**	**(0.34)**
**Excited state 4**				
307 nm (*f* =0.0837)	**159 (HOMO)**	⟶	**161 (LUMO+1)**	**(0.56)**
	159 (HOMO)	⟶	165 (LUMO+6)	(0.18)

S5.4 TD-DFT UV/Vis absorption spectra of MIC-Ir(III) relevant complexes.

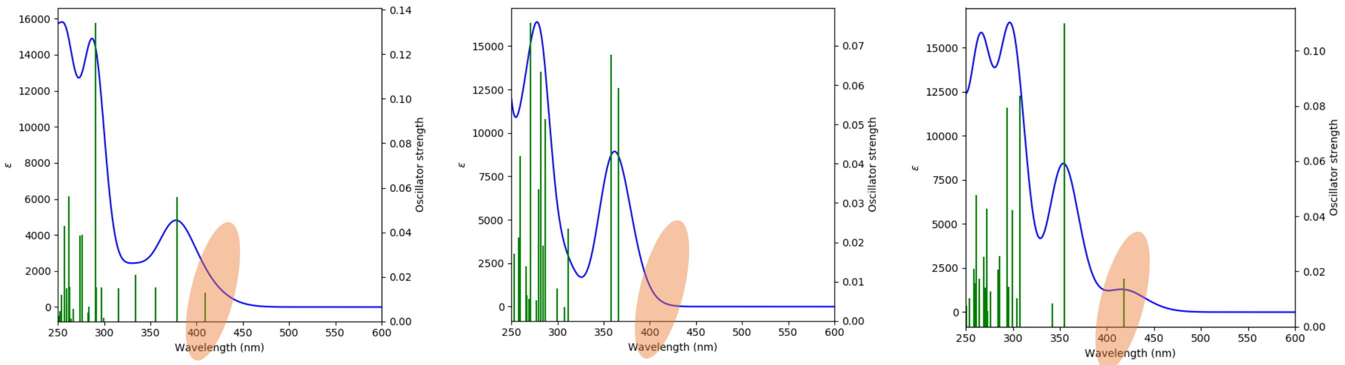

Complex **I** Complex **II** Complex **Ir-H**

Figure S22 TD-DFT calculated UV-VIS absorbance spectrum of iridium complexes. A new absorption band is predicted at 420 nm for the **Ir-H** complex.

S5.5 Experimental absorption, excitation, and emission spectra of iridium complexes

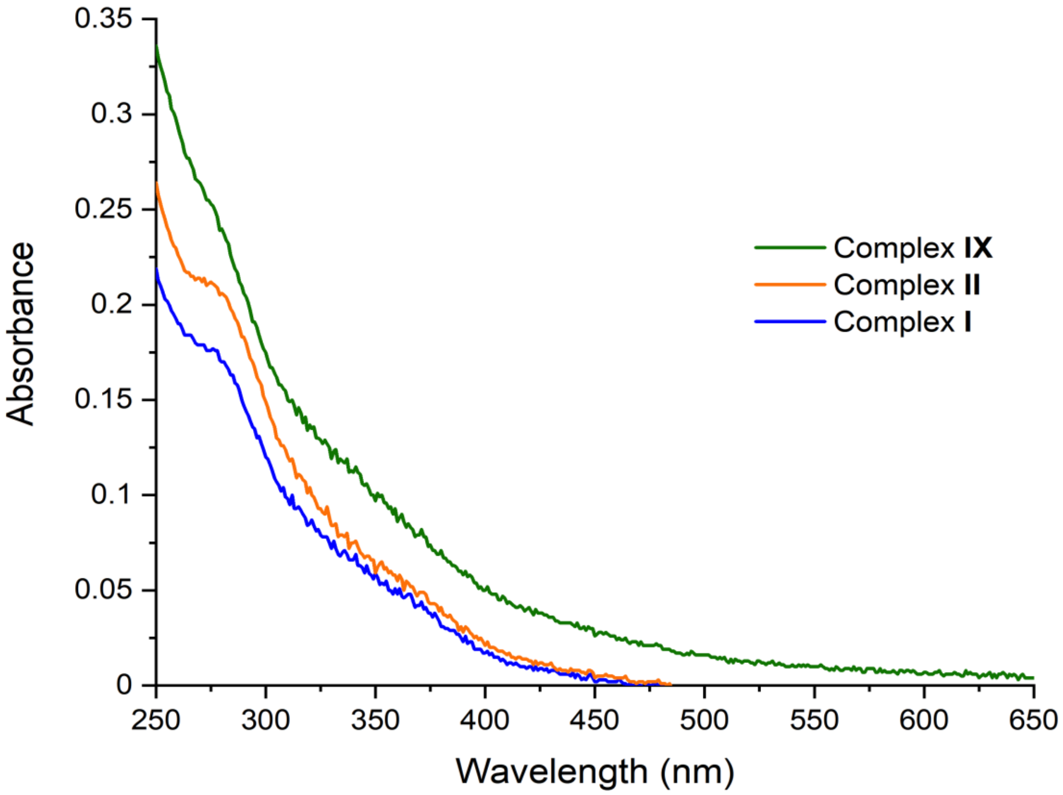

Figure S23 Observed absorption spectra of complexes **I, II** and **IX** (1.0 x 10^{-5} M in degassed methanol).

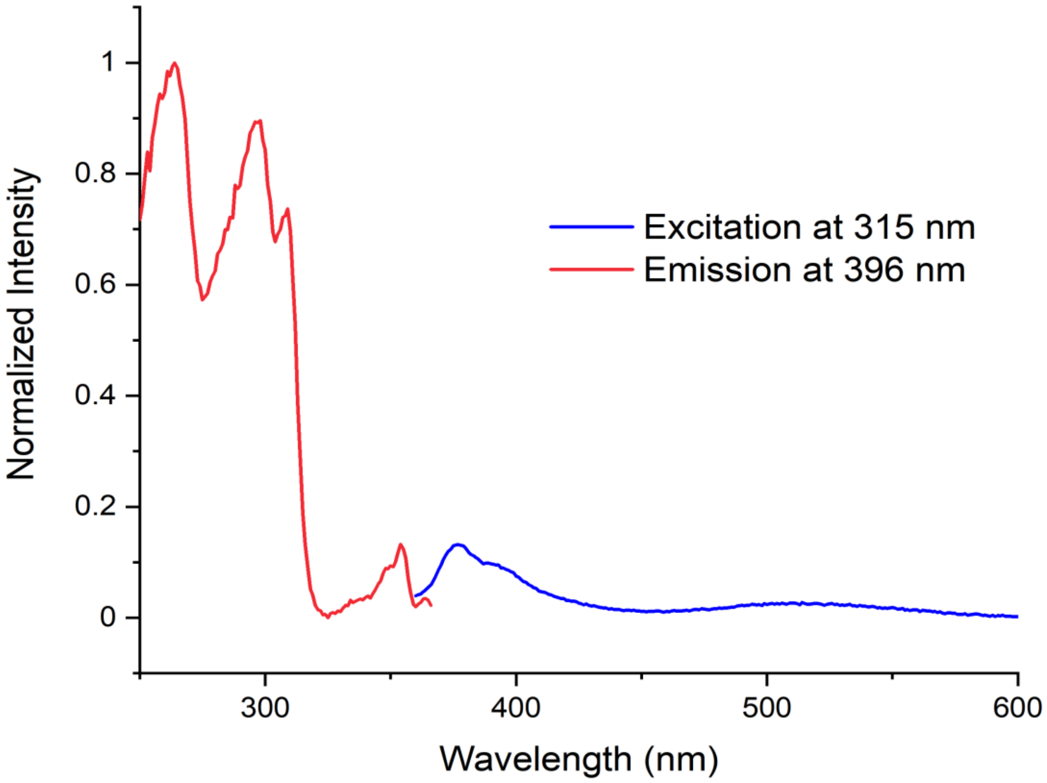

Figure S24 Observed emission and excitation spectra of a solution of complex **II** at 298 K in methanol.

S5.6 Comparison of absorption spectra of complexes overlaid with emission spectra of LED lamps.

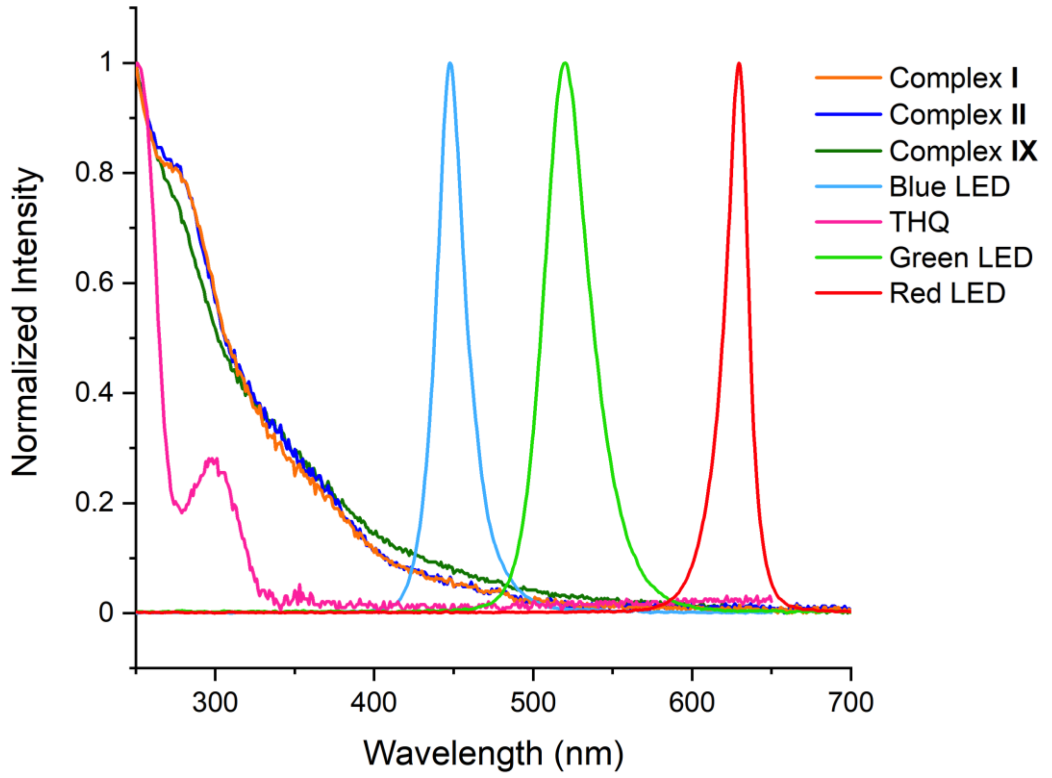

Figure S25 UV-VIS absorbance spectra of complexes **I**, **II, IX** and THQ (1.0 x 10^{-5} M in degassed methanol) overlaid with the emission spectra of the blue, green and red LED lamps.

S6 Thermal dehydrogenation of N-heterocycles

Catalytic experiments were performed in a 25 mL Schenk flask connected to a condenser containing a bubbler filled with mineral oil to exclude air from the reaction system and to allow the release of molecular hydrogen.

Table S9 Thermal dehydrogenation of N-heterocycles under various reaction conditions.

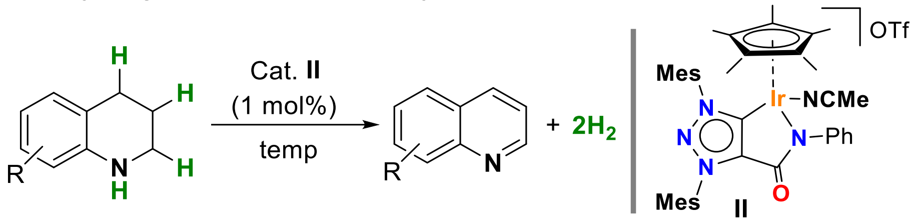

Entry	Subs.	Prod.	Cat.(mol%)	T (°C)	Solvent	[Subs]	Yield (%)
1	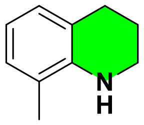	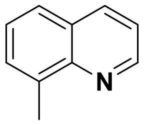	1	30	MeOH	0.25 M	0
2			1	80	MeOH	0.25 M	0
3			2	30	MeOH	0.1 M	0
4			2	80	MeOH	0.1 M	0
5	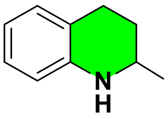	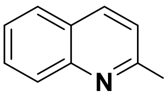	1	120	CH_3CN	0.25 M	4
6			1	30	Toluene	0.25 M	0
7			2	30	Toluene	0.1 M	0
8	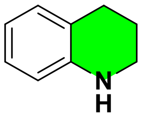	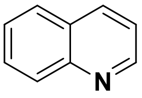	1	130	Toluene	0.25 M	88
9	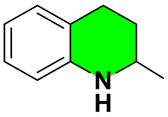	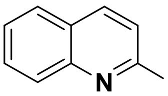	1	130	Toluene	0.25 M	90
10[a]			1	130	Toluene	0.25 M	0
11[b]	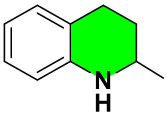	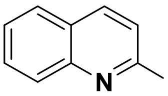	1	130	Toluene	0.25 M	0
12	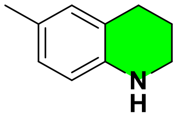	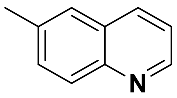	1	130	Toluene	0.25 M	80
13			2	130	Toluene	0.1 M	70
14	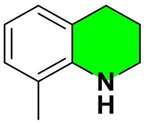	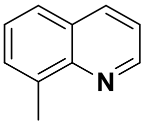	1	130	Toluene	0.25 M	92
15	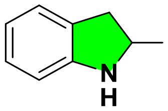	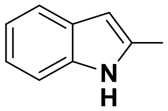	1	130	Toluene	0.25 M	93

Reaction conditions: substrate (0.5 mmol), catalyst, solvent for 18 h. Product formation (yield) obtained by GC/FID using hexadecane as an internal standard. [a] Reaction without catalyst. [b] Reaction carried out in a closed system.

S7 Experimental evidence of hydride formation

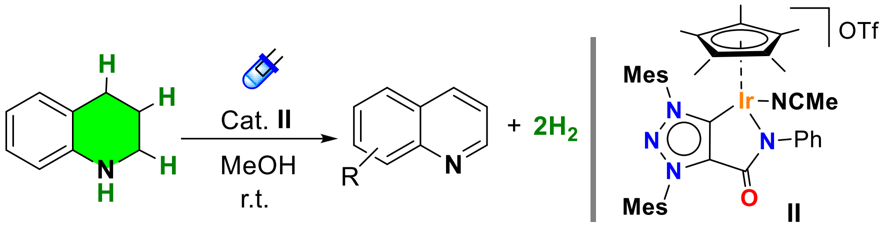

Under inert atmosphere, an NMR tube with a Teflon-lined cap was charged with 5 mg (0.0053 mmol) of catalyst **II**, deoxygenated methanol-d4 (1 mL) and 5μL (0.04 mmol) of 1,2,3,4-tetrahydroquinoline (THQ). Reaction mixture was irradiated at room temperature, with blue LEDs (λ 455 nm) for 3h and monitored by ^{1}H NMR in a closed system.

^{1}H NMR spectra at selected times (0 and 3 h) show the evolution of 1,2,3,4-tetrahydroquinoline in presence of catalyst **II** under inert conditions (Figure S26). After irradiation with blue LED light, a new signal appears at -14.23 ppm, typical of hydride species. In addition, it is also characteristic the disappearance the Cp* signal of complex **II** at 1.13 ppm, while the appearance of a new Cp* signal of Ir-H complex at 1.35 ppm (highlighted in blue). Both remarks confirm the presence of a hydride complex as intermediate during the catalytic transformation as resting state.

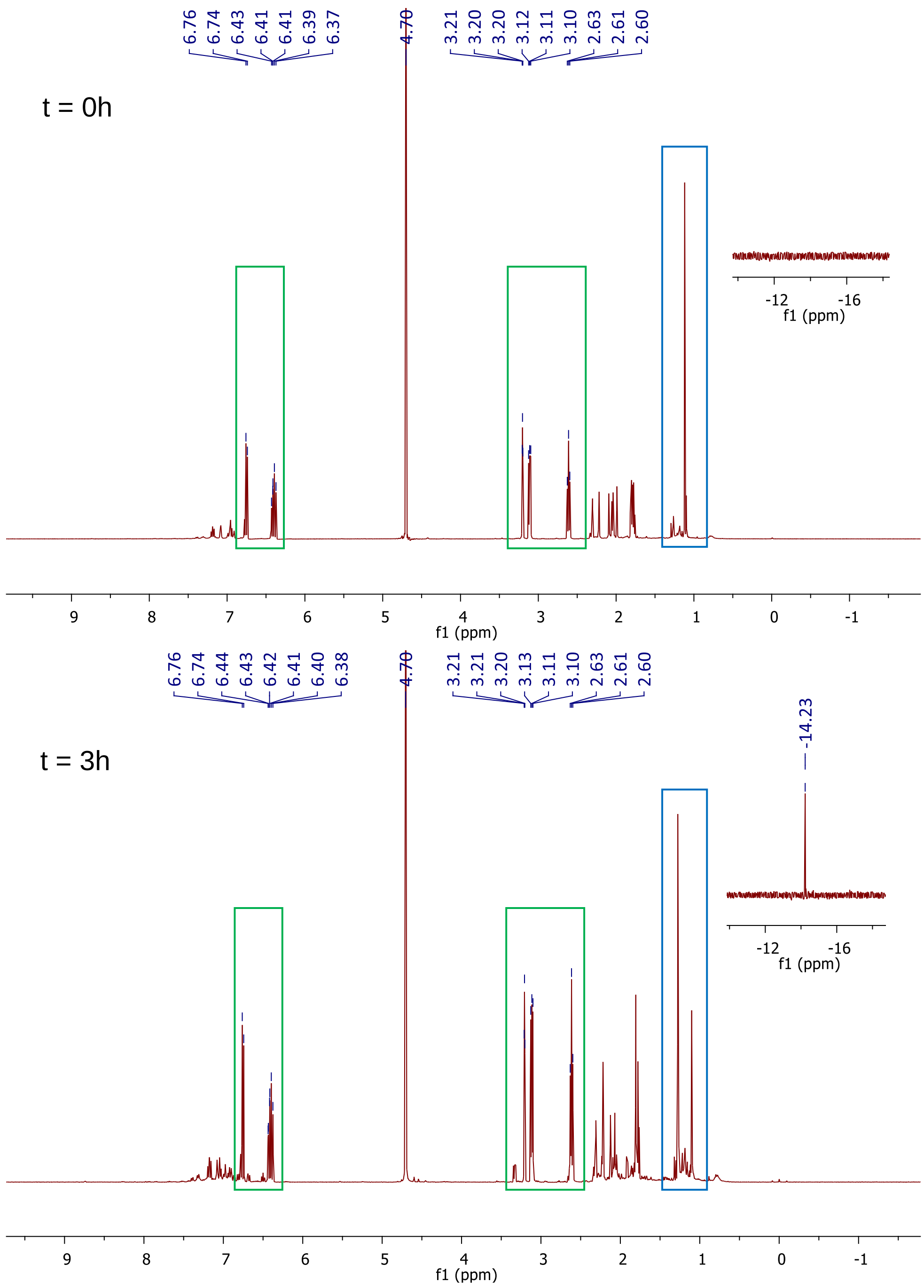

Figure S26 ^{1}H NMR spectra showing the formation of an Ir-H in the photocatalytic reaction of THW in the presence of complex **II** using methanol-d4 (residual solvent signal 4.70 ppm). Highlighted signals: Cp* group (blue) and 1,2,3,4-tetrahydroquinoline (green)

S8 Experimental evidence of dehydrogenation sequence in N-heterocycles induced by visible light

The double photodehydrogenation of N-heterocycles in THQs may happen at the NH-C(2)H (imine intermediate) or at the C(3)H-C(4)H remote position. In order to stablish the dehydrogenation sequence, we used a model compound where dehydrogenation next to N is blocked and analyzed the possibility of direct dehydrogenation at C(3)H-C(4)H. Using catalysts **II** under 18h irradiation, we have observed that dehydrogenation of C(3)H-C(4)H does not occur. This result suggests that dehydrogenation sequence in N-heterocycles is produced via a first dehydrogenation next to N, tautomerization and second dehydrogenation.

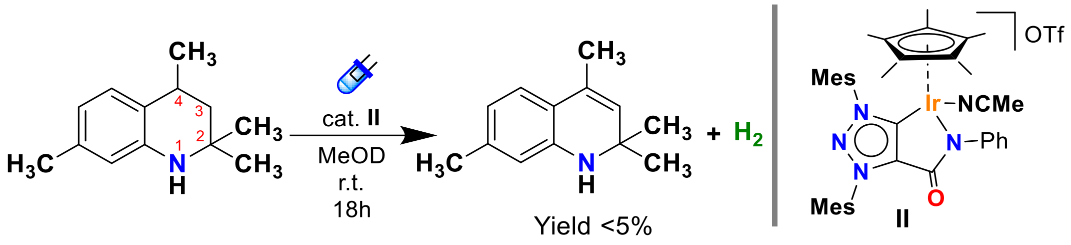

Figure S27 Photodehydrogenation of 1,2,3,4-tetrahydro-2,2,4,7-tetramethylquinoline.

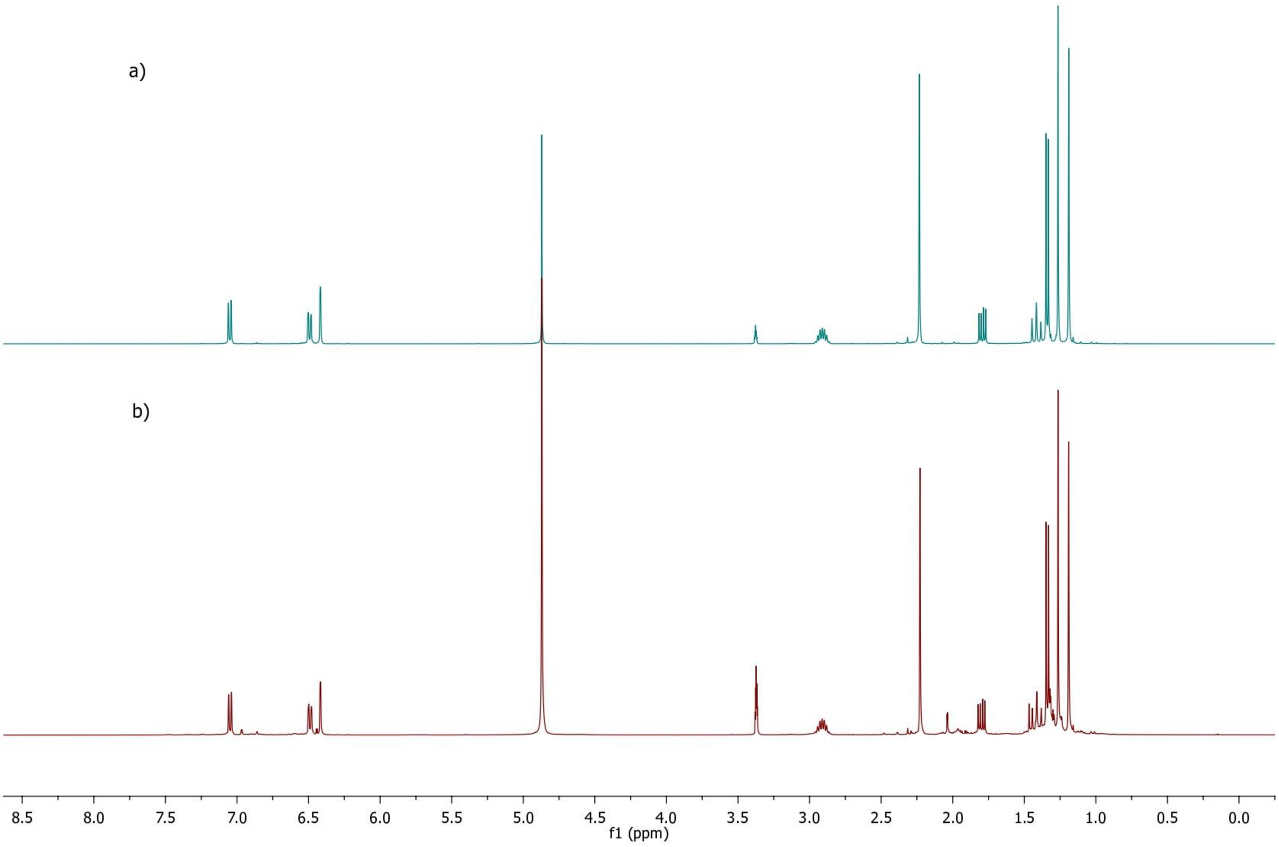

Figure S28 ^{1}H NMR spectra in photodehydrogenation of 1,2,3,4-tetrahydro-2,2,4,7-tetramethylquinoline in methanol-d4 (residual solvent signals at 4.70 and 3.35 ppm) at t = 0h (a) and after t = 18h (b).

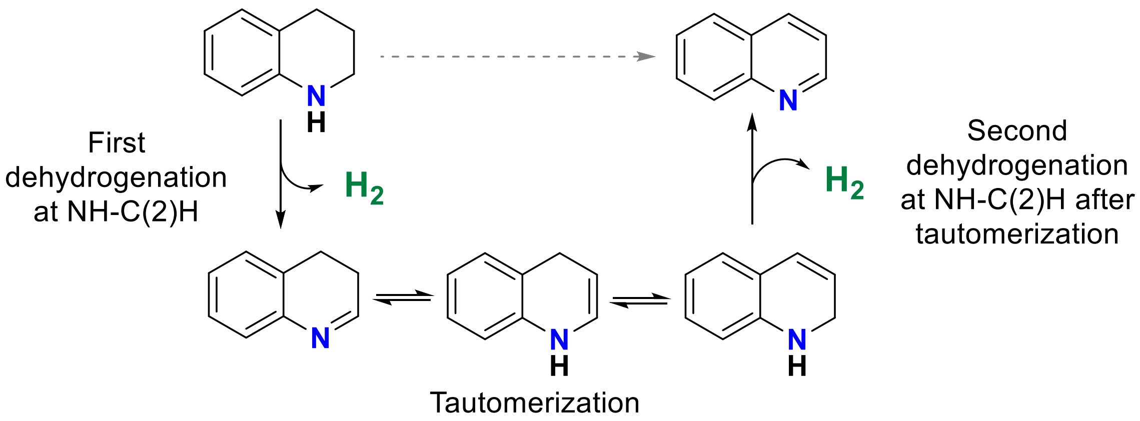

Figure S29 Proposed double dehydrogenation sequence of N-heterocycles.

S9 Complete mechanistic proposal

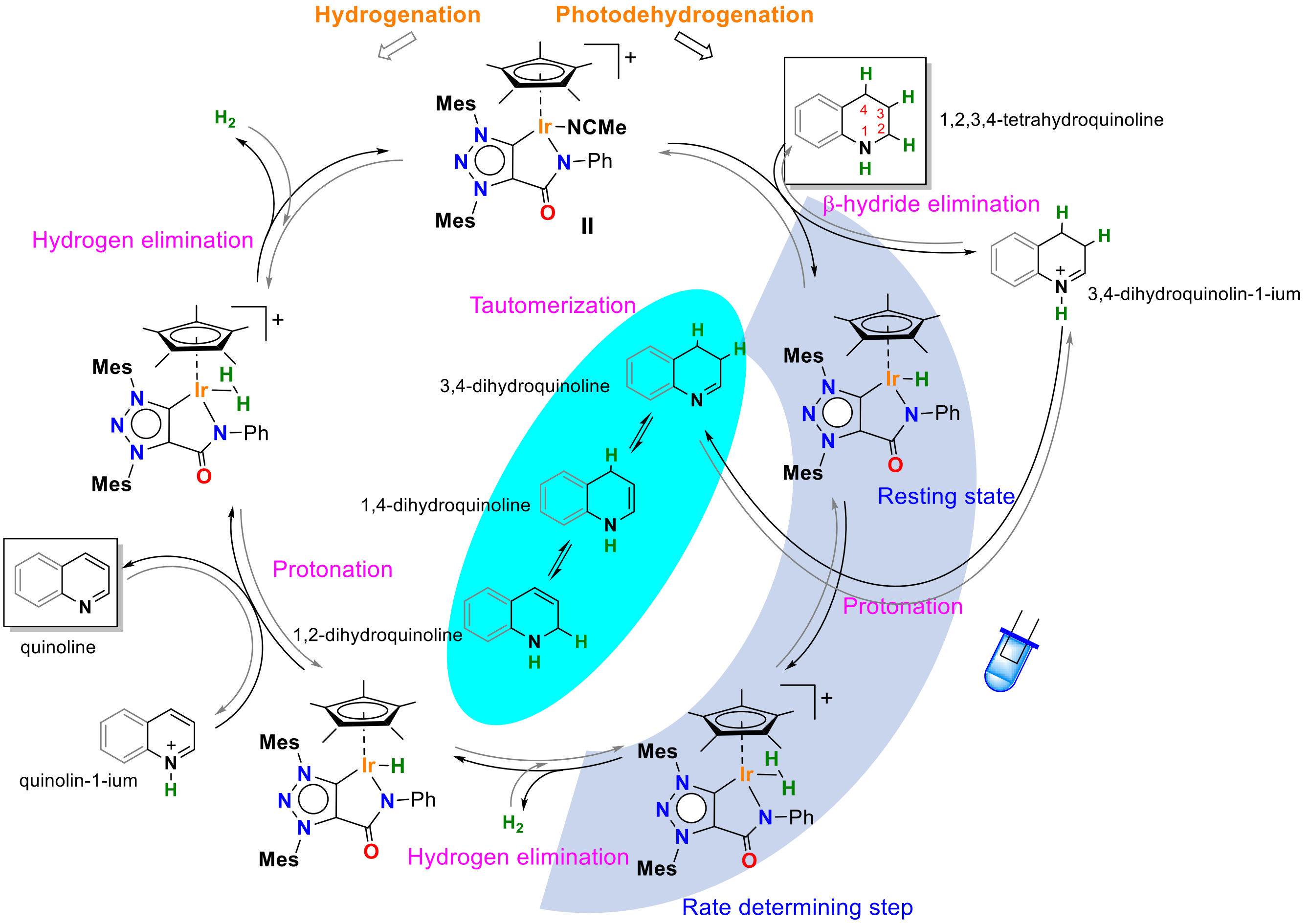

Figure S30 Complete mechanistic proposal showing photodehydrogenation and hydrogenation pathways.

S10 Hydrogenation of N-heterocycles

All high-pressure catalyzed hydrogenation reactions were carried out in a 100 mL stain steel autoclave. Catalytic reactions were carried out in 2 mL GC glass vials, which were set in an alloy plate and placed inside the autoclave. The autoclave was purged with 10 bar of hydrogen for three times before setting the pressure to the desired value. Each GC glass vial was loaded with the corresponding substrate (0.2 mmol), catalyst (1 mol %) and dry/deoxygenated toluene (1 mL). The autoclave was loaded with 2.5 bar of H_2, sealed and heated using a hot plate at 80ºC for 6h. Conversions and yields were determined by GC relative to hexadecane as internal standard and by ^{1}H NMR spectroscopy.

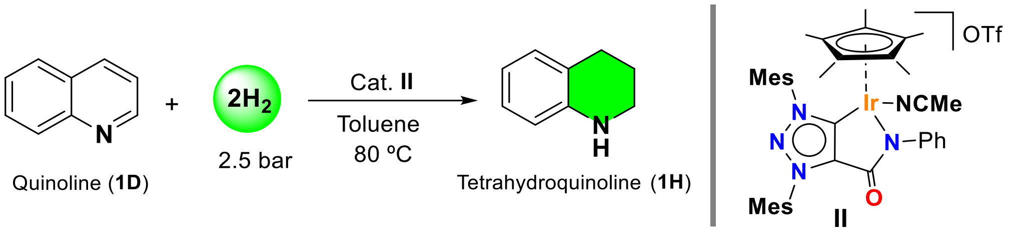

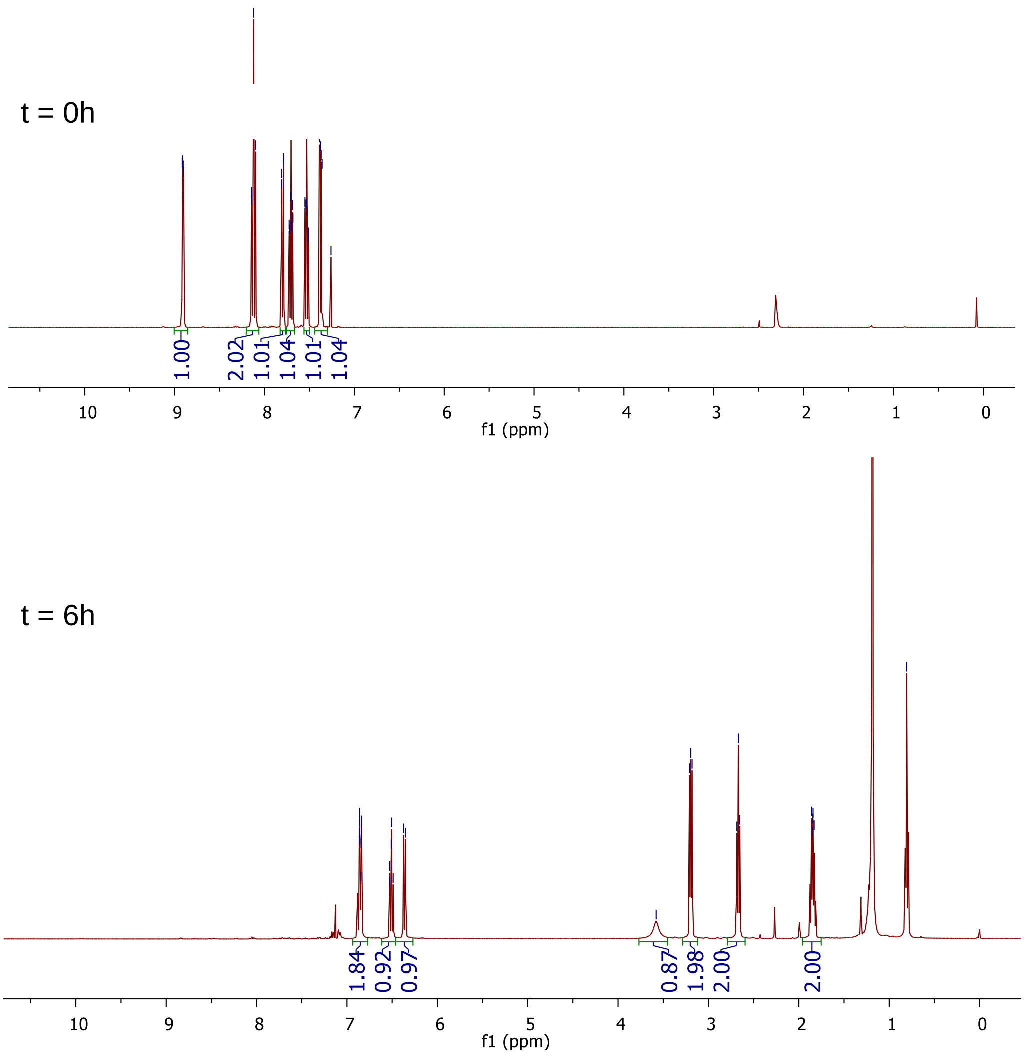

Figure S31 Hydrogenation of **1D**. ^{1}H NMR spectrum at t = 0h and t = 6h reaction using hexadecane (signals at 1.26 and 0.88 ppm) as an internal standard. Residual $CDCl_3$ solvent signal at 7.26 ppm.

S11 References

(1) Coppola, G. M.; Damon, R. E. Acetylenic Amides. I. Synthesis of N-Substituted-2-Propynamides. *Synth. Commun.* **1993**, *23* (14), 2003–2010. https://doi.org/10.1080/00397919308009860.

(2) Bouffard, J.; Keitz, B. K.; Tonner, R.; Guisado-Barrios, G.; Frenking, G.; Grubbs, R. H.; Bertrand, G. Synthesis of Highly Stable 1,3-Diaryl-1 H -1,2,3-Triazol-5-Ylidenes and Their Applications in Ruthenium-Catalyzed Olefin Metathesis. *Organometallics* **2011**, *30* (9), 2617–2627. https://doi.org/10.1021/om200272m.

(3) Mintz, M. J.; Walling, C. ^{t}Butyl Hypochlorite. *Org. Synth.* **1969**, *49* (September), 9. https://doi.org/10.15227/orgsyn.049.0009.

(4) Li, Q.; Zhang, S.-Y.; He, G.; Ai, Z.; Nack, W. A.; Chen, G. Copper-Catalyzed Carboxamide-Directed Ortho Amination of Anilines with Alkylamines at Room Temperature. *Org. Lett.* **2014**, *16* (6), 1764–1767. https://doi.org/10.1021/ol500464x.

(5) Almodares, Z.; Lucas, S. J.; Crossley, B. D.; Basri, A. M.; Pask, C. M.; Hebden, A. J.; Phillips, R. M.; McGowan, P. C. Rhodium, Iridium, and Ruthenium Half-Sandwich Picolinamide Complexes as Anticancer Agents. *Inorg. Chem.* **2014**, *53* (2), 727–736. https://doi.org/10.1021/ic401529u.

(6) Beppu, T.; Sakamoto, K.; Nakajima, Y.; Matsumoto, K.; Sato, K.; Shimada, S. Hydrosilane Synthesis via Catalytic Hydrogenolysis of Halosilanes Using a Metal-Ligand Bifunctional Iridium Catalyst. *J. Organomet. Chem.* **2018**, *869*, 75–80. https://doi.org/10.1016/j.jorganchem.2018.05.024.

(7) Sypaseuth, F. D.; Matlachowski, C.; Weber, M.; Schwalbe, M.; Tzschucke, C. C. Electrocatalytic Carbon Dioxide Reduction by Using Cationic Pentamethylcyclopentadienyl-Iridium Complexes with Unsymmetrically Substituted Bipyridine Ligands. *Chem. - A Eur. J.* **2015**, *21* (17), 6564–6571. https://doi.org/10.1002/chem.201404367.

(8) Petronilho, A.; Rahman, M.; Woods, J. A.; Al-Sayyed, H.; Müller-Bunz, H.; Don MacElroy, J. M.; Bernhard, S.; Albrecht, M. Photolytic Water Oxidation Catalyzed by a Molecular Carbene Iridium Complex. *Dalt. Trans.* **2012**, *41* (42), 13074–13080. https://doi.org/10.1039/c2dt30403a.

(9) Xiao, X.-Q.; Jin, G.-X. Functionalized N-Heterocyclic Carbene Iridium Complexes: Synthesis, Structure and Addition Polymerization of Norbornene. *J. Organomet. Chem.* **2008**, *693* (21–22), 3363–3368. https://doi.org/10.1016/j.jorganchem.2008.08.019.

(10) Sabater, S.; Baya, M.; Mata, J. A. Highly Active Cp*Ir Catalyst at Low Temperatures Bearing an N-Heterocyclic Carbene Ligand and a Chelated Primary Benzylamine in Transfer Hydrogenation. *Organometallics* **2014**, *33* (23), 6830–6839. https://doi.org/10.1021/om500888d.

(11) Strydom, I.; Guisado-Barrios, G.; Fernández, I.; Liles, D. C.; Peris, E.; Bezuidenhout, D. I. A Hemilabile and Cooperative N-Donor-Functionalized 1,2,3-Triazol-5-Ylidene Ligand for Alkyne Hydrothiolation Reactions. *Chem. - A Eur. J.* **2017**, *23* (6), 1393–1401. https://doi.org/10.1002/chem.201604567.

(12) Frisch, M. J.; Trucks, G. W.; Schlegel, H. B.; Scuseria, G. E.; Robb, M. A.; Cheeseman, J. R.; Scalmani, G.; Barone, V.; Mennucci, B.; Petersson, G. A.; Nakatsuji, H.; Caricato, M.; Li, X.; Hratchian, H. P.; Izmaylov, A. F.; Bloino, J.; Zheng, G.; Sonnenberg, J. L.; Hada, M.; Ehara, M.; Toyota, K.; Fukuda, R.; Hasegawa, J.; Ishida, M.; Nakajima, T.; Honda, Y.; Kitao, O.; Nakai, H.; Vreven, T.; Montgomery, J. A.; Peralta, J. E.; Ogliaro, F.; Bearpark, M.; Heyd, J. J.; Brothers, E.; Kudin, K. N.; Staroverov, V. N.; Kobayashi, R.; Normand, J.; Raghavachari, K.; Rendell, A.; Burant, J. C.; Iyengar, S. S.; Tomasi, J.; Cossi, M.; Rega, N.; Millam, J. M.; Klene, M.; Knox, J. E.; Cross, J. B.; Bakken, V.; Adamo, C.; Jaramillo, J.; Gomperts, R.; Stratmann, R. E.; Yazyev, O.; Austin, A. J.; Cammi, R.; Pomelli, C.; Ochterski, J. W.; Martin, R. L.; Morokuma, K.; Zakrzewski, V. G.; Voth, G. A.; Salvador, P.; Dannenberg, J. J.; Dapprich, S.; Daniels, A. D.; Farkas; Foresman, J. B.; Ortiz, J. V; Cioslowski, J.; Fox, D. J. Gaussian 09, Revision B.01. *Gaussian 09, Revision B.01, Gaussian, Inc., Wallingford CT*. 2010.
